# Lifestyle physical activity coaching in outpatients with major depressive disorder (PACOUTPAT): study protocol for a randomized controlled trial on physical activity, depression, and quality of life

**DOI:** 10.1186/s13063-026-09500-1

**Published:** 2026-02-17

**Authors:** Céline Stark, Johannes Beck, Anja Oswald, Anja Rogausch, Ann-Katrin Schreiner, Robyn Cody, Vivien Hohberg, Florian Knappe, Jan-Niklas Kreppke, Sebastian Ludyga, Markus Gerber

**Affiliations:** 1 University of Basel, Basel, Switzerland; 2 Psychiatric Clinic Sonnenhalde, Riehen, Switzerland; 3 University of Zurich, Zurich, Switzerland; 4 Department of Sport, Exercise and Health, University of Basel, Grosse Allee 6, Basel, CH-4052 Switzerland

**Keywords:** Affect, Behavior change, Coaching, Depression, Lifestyle, Motivation, Outpatients, Physical activity, Psychiatry, Wearable

## Abstract

**Background:**

Major depressive disorder (MDD) is highly prevalent and associated with substantial disease burden and significantly reduced life expectancy. Physical activity (PA) has been shown to reduce depressive symptoms to a similar extent as antidepressant medication. Despite this evidence, individuals with MDD engage in lower levels of PA and exhibit higher levels of sedentary behavior. Structural, physical, and psychological barriers hinder PA engagement. PA coaching offers a promising strategy to overcome these barriers and promote sustainable behavior change. Previous research highlights the importance of addressing affective responses to PA in interventions. Moreover, the support of technology-based tools, such as wearables, seems promising. This study aims to investigate the short- and long-term effects of a new PA coaching approach on PA behavior, depression severity, and quality of life in outpatients with MDD. Potential mechanisms of behavior change and participant experiences are also examined.

**Methods:**

This study uses a three-arm randomized controlled trial (RCT) design and is conducted at a psychiatric day clinic in German-speaking Switzerland. A total of 114 individuals aged 18 to 65 years with a clinical diagnosis of MDD (ICD-10: F32 and F33) are recruited on an ongoing basis and randomly assigned to one of three groups: (1) A 10-week PA coaching program based on interest and experience in PA, supported by a wearable smart ring for self-monitoring; (2) the same 10-week program extended by an additional, remote 26-week cognition-based coaching phase; or (3) a control group receiving treatment as usual along with written current PA guidelines. Data is collected at three measurement timepoints: 2-4 weeks after the start of day clinic treatment (baseline), after the 10-week coaching phase (post), and after a 6-month follow-up period (follow-up). The primary outcome is the change in device-measured PA over time (baseline, post, and follow-up), measured via Fibion^®^ accelerometer, and analyzed using linear mixed models.

**Discussion:**

A new PA coaching program is introduced to counteract low PA levels observed in individuals with MDD. By combining affective with cognitive components of behavior change, the intervention aims to promote a sustainable increase in PA and ultimately improve mental and physical health and quality of life.

**Trial registration:**

DRKS: DRKS00036209. Registered on 08 May 2025

**Supplementary Information:**

The online version contains supplementary material available at 10.1186/s13063-026-09500-1.

## Administrative information

**Table:** 

Title {1}	Lifestyle physical activity coaching in outpatients with major depressive disorder (PACOUTPAT): study protocol for a randomized controlled trial on physical activity, depression and quality of life
Trial registration {2a and 2b}	DRKS00036209
Protocol version {3}	Version 2, 25.04.2025
Funding {4}	The study is funded by the University of Basel and financially supported by the Freie Akademische Gesellschaft (FAG) with a contribution of CHF 30,000, which is used to pay the physical activity coaches.
Author details {5a}	^1^University of Basel, Basel, Switzerland ^2^Psychiatric Clinic Sonnenhalde, Riehen, Switzerland ^3^University of Zurich, Zurich, Switzerland
Name and contact information for the trial sponsor {5b}	Markus Gerber, PhD, Department of Sport, Exercise and Health, University of Basel Grosse Allee 6, CH-4052 Basel, Switzerland Email: markus.gerber@unibas.ch, Tel.: +41 61 20 747 83; Fax: +41 61 20 747 89
Role of sponsor {5c}	Sponsor and co-principal investigator are the same person (MG). The sponsor is responsible for the overall implementation of the project, including project management, supervision of study personnel, and adherence to quality management procedures. The sponsor holds responsibility for the design of the trial, data collection, management and analyses. The sponsor also coordinates the development and execution of publication plan and dissemination of results. The funders – University of Basel and Freie Akademische Gesellschaft (FAG) Basel – have no influence on trial design, data collection and analyses and publication plan.

## Introduction

### Background and rationale {6a}

Major depressive disorder (MDD) is among the most prevalent mental health conditions worldwide, affecting an estimated 332 million people in 2021, corresponding to a global prevalence of 4% [1] and a lifetime prevalence of 11% [2]. In 2022, disability-adjusted life years (DALYs) attributable to MDD reached 50 million, with projections indicating an increase to 68 million by 2050 [3]. A key factor in the disabling nature of MDD is its negative impact on health-related quality of life [4]. Beyond impairing daily functioning and overall well-being, MDD generates considerable economic costs. Indirect costs — such as household expenses, presenteeism, and absenteeism — are about as high as direct healthcare costs [5]. Individuals with severe mental illness, including MDD, face a 2- to 3.5-fold increase in all-cause mortality risk compared to the general population [6]. Accordingly, individuals with severe mental illnesses experience a 20% reduction in life expectancy and are estimated to die, on average, 10 to 25 years earlier than the general population [7–11]. While elevated mortality from unnatural causes such as suicide, accidents, and substance misuse contributes significantly to this disparity [7, 12, 13], the majority of excess deaths are due to natural causes, particularly cardiovascular disease, respiratory conditions, and infectious diseases [14–20].

Given the high prevalence and substantial burden of MDD, identifying effective treatment strategies is of critical importance [21]. Current clinical treatment guidelines recommend psychotherapy and/or pharmacological antidepressants as first-line treatments for MDD [22, 23]. However, antidepressant use is frequently accompanied by side effects — such as nausea, weight gain, incontinence, and sexual dysfunction [24, 25] — as well as low adherence rates [26] and limited efficacy [27, 28]. Approximately, 30% of patients do not respond to first-line treatment, and even after medication switches or augmentation, persistent non-response and adverse effects remain a major clinical challenge [29–31].

In light of these limitations of standard care, exercise has gained increasing attention as a non-pharmacological approach to alleviating depressive symptoms. Compelling evidence suggests that physical activity (PA) interventions produce moderate to large antidepressant effects [32, 33]. The effect of exercise has even been demonstrated to be comparable to antidepressant medication [34], as confirmed by several meta-analyses [35–37]. Furthermore, regular exercise following the treatment period has been found to decrease the rate of relapse by 50% after 6 months [38]. Beyond its antidepressant effects, PA has also been associated with improvements in broader health outcomes, including health-related quality of life [39], cardiovascular risk [21, 40], cognition [41], sleep [42], and potential biomarkers of depression such as cortisol levels [43]. Owing to the well-documented benefits of exercise in the treatment of depression, PA is now recommended as an effective complementary treatment modality in several national and international clinical guidelines [44–48].

Despite strong evidence supporting exercise as a treatment for depression, its inclusion in clinical guidelines, and its adoption by an increasing number of clinics as a standard therapy [49, 50], PA levels among adults with depression remain significantly lower than in the general population. Data indicate that approximately 86% of individuals with depression do not meet recommended PA levels and tend to engage in more sedentary behavior compared to people without depression [51]. Explanations for the discrepancy between the well-established efficacy of PA and actual PA behavior can, on the one hand, be identified within clinical practice: although PA programs are available in almost all psychiatric hospitals in Switzerland, only about one in four patients utilize them [49]. This underutilization reflects a broader trend of PA being insufficiently prescribed to individuals with MDD [49]. Key contributing factors include limited training among treatment personnel, skepticism regarding patient adherence, and a lack of collaboration with exercise specialists [52].

On the other hand, at the individual level, people with depression face both physical and psychological barriers to engaging in regular PA. Common barriers include (higher levels of) depression symptoms, higher body mass index (BMI), and the presence of somatic comorbidities [53]. In addition, motivational and volitional deficits in all areas of daily life interfere with the aim of engaging depressed patients in more exercise and PA. Individuals with MDD demonstrate fewer action plans, have less maintenance self-efficacy, and are more easily distracted by barriers [54, 55]. These challenges highlight the need for targeted interventions that provide patients with professional support to enable them to overcome barriers and adopt a physically active lifestyle [53, 56, 57]. In addition, it is important to put effort into the promotion of behavioral skills necessary for patients to build up and maintain PA [46, 56].

Given this background, PA coaching has proven to be feasible and well accepted in inpatients with MDD, as demonstrated in a recent RCT [58, 59]. Despite the feasibility and acceptance, no long-term effects on PA behavior were observed [60]. A qualitative evaluation of the intervention identified several participant-reported challenges, including limited contact during predominantly remote coaching sessions held at 2-week intervals and the absence of structured support following the intervention period [61]. These findings highlight the need for strategies that foster sustained PA behavior beyond the duration of the coaching itself.

PA coaching in PACINPAT was based on the motivation-volition (MoVo) process model [62] and behavior change techniques [63], both grounded in social-cognitive approaches to behavior change. Recent advances in exercise psychology emphasize the role of affective as well as cognitive determinants of behavior [64–68]. The Affective-Reflective Theory of physical inactivity and exercise (ART) distinguishes two coexisting processes: an automatic affective valuation and a reflective cognitive process [64]. In MDD, diminished motivation and volition may reduce the impact of the cognitive reflective process on activity decisions [54, 64], making the enhancement of positive affective responses to PA a promising strategy for both initiating and maintaining long-term participation in PA [69–72].

In addition, emerging technologies, such as wearables, offer substantial potential for supporting exercise behavior change [73–75]. Integrating such technologies into future coaching interventions may enhance monitoring of PA behavior for both patients and coaches. Moreover, wearables can support the sustainability of intervention effects by enabling self-monitoring to continue after the formal coaching has ended [76].

## Objectives {7}

Building on the background outlined above, the primary objective of this study is to examine the short- and long-term effects of a novel PA coaching program to promote PA behavior in outpatients with MDD receiving treatment as usual. The intervention consists of a structured PA coaching approach that integrates both affective (interest- and experience-based) and cognitive (reflective) strategies to facilitate the adoption of a sustainable PA routine. To enhance engagement and adherence, participants are also supported through continuous self-monitoring using a commercially available smart ring.

Secondary objectives are (i) to assess short- and long-term effects of the PA coaching program on quality of life and depression severity and (ii) to investigate potential mechanisms responsible for the targeted behavior change.

## Trial design {8}

This study is a single-center, three-arm randomized controlled trial (RCT) with an allocation ratio of 1:1:1. It includes two intervention groups (IG1, IG2) and one control group (CG) and follows an open-label design. The trial is conducted in collaboration between the Sonnenhalde AG Psychiatric Clinic, Riehen, Switzerland, and the Department of Sport, Exercise and Health (DSBG) at the University of Basel, Basel, Switzerland.

The intervention is delivered in an outpatient setting, recruiting patients from day clinics of the Sonnenhalde AG. Assessments are conducted at three time points: (a) baseline (T0), (b) post-intervention (T1, 10 weeks after baseline), and (c) follow-up (T2, 36 weeks after baseline). The follow-up period of 26 weeks after post-intervention ensures alignment with prior comparable studies [77–79]. All assessments are conducted on-site at the day clinics.

**Fig. 1:**
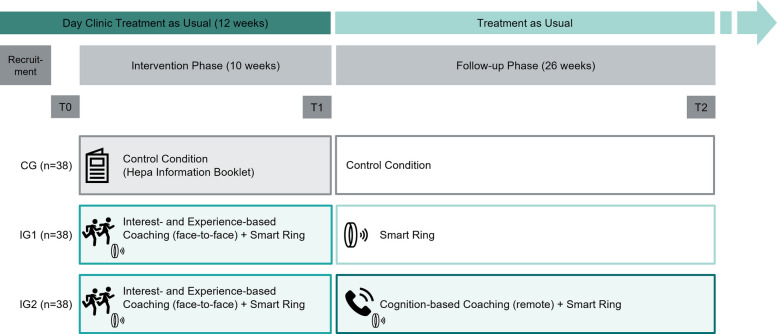
Overview of the study design

An overview of the study design is provided in Fig. 1. The protocol is designed in accordance with the SPIRIT 2025 statement for interventional trials [80].

Participants (*N* = 114) are randomized (stratified by sex and age) to one of two PA coaching conditions (with or without continuous support after post-intervention) or to a control condition (written information on health-enhancing PA) [81]. Given the behavioral nature of the intervention, neither participants nor researchers are blinded. To reduce assessment bias, the primary outcome (PA behavior) is assessed device-based using a thigh-worn accelerometer device.

The trial features several pragmatic elements [82–84], such as integration into treatment as usual, delivery in real-world and authentic settings (e.g., home-based components), and use of a commercially available smart ring for self-monitoring. Coaching protocols are adapted for clinical populations, and outcomes focus on clinically relevant indicators, including depressive symptom severity and quality of life.

## Methods: participants, interventions, and outcomes

### Study setting {9}

The study is conducted from summer 2025 until autumn 2027 at day clinics of the Sonnenhalde AG Psychiatric Clinic by the Department of Sport, Exercise and Health (DSBG) of the University of Basel.

### Eligibility criteria {10}

Participants are eligible for participation in the study if they are 18 to 65 years old, are receiving outpatient treatment at the day clinic, and are diagnosed with a depressive disorder (F32, F33) by a physician. Further inclusion criteria are sufficient German language skills, signed informed consent, and voluntary participation.

Exclusion criteria are medical contraindications to PA, as assessed by a physician or indicated by the Physical Activity Readiness Questionnaire (PAR-Q) [85], current psychosis and/or acute suicidality, and pregnancy or breastfeeding at the time of assessment. In cases where medical contraindications are initially present, participants may still be enrolled if they provide written confirmation from their general practitioner (or from another physician) stating that participation in PA is medically acceptable.

### Who will take informed consent? {26a}

At the beginning of their day clinic treatment, patients receive an information folder including a flyer about the PACOUTPAT study. Study personnel from the University of Basel are present 2 days per week, and interested patients are invited to a one-on-one meeting with a study team member, where detailed oral and written information is provided. The researcher explains the study’s purpose, procedures, duration, potential risks and benefits, and the voluntary nature of participation. It is emphasized that withdrawal from the study is possible at any time without negative consequences. Patients receive a participant information sheet and an informed consent form. They are given up to 1 week to ask questions or request further details before deciding. Written informed consent is obtained prior to any study procedures.

### Additional consent provisions for collection and use of participant data and biological specimens {26b}

Not applicable as no further use of participant data is planned and no biological specimens are collected.

## Interventions

### Explanation for the choice of comparators {6b}

By comparing PA coaching interventions (IG1 and IG2) with standard treatment (CG), this study assesses the effectiveness and potential superiority of PA coaching in increasing PA, alleviating depressive symptoms, and enhancing quality of life. Furthermore, the comparison between a 10-week coaching intervention (IG1) and an extended version that includes an additional 6 months of support (IG2) provides insights into the impact of coaching duration on the sustainability of PA engagement, depression severity, and health-related quality of life. The inclusion of standard treatment across all groups ensures a pragmatic, real-world approach, allowing for a meaningful evaluation of the added benefits of PA coaching within routine clinical care.

### Intervention description {11a}

Participants in both intervention groups continue to receive treatment as usual according to the Swiss treatment guidelines for MDD [86]. The intervention is designed to provide individually tailored support and encouragement to increase PA during and after clinical outpatient treatment for a period of up to 36 weeks (= 10 + 26 weeks).

### Intervention Group 1

Participants in IG1 receive a 10-week PA coaching program guided by a trained personal PA coach. The coaching follows an interest- and experience-based approach aimed at identifying PA opportunities that align with participants’ individual preferences and promote positive affective experiences. Where appropriate, structured PA is preferred over unstructured forms to leverage the benefits of group-based and/or supervised exercise, such as increased social support and accountability. The coaching approach is individualized and flexible, supporting unstructured PA when it better matches the participant’s interests and enjoyment. The primary focus is on promoting pleasure and well-being during PA with the ultimate goal of facilitating long-term adherence.

The coaching begins with a 60-min introductory session during which the coach and participant get to know each other, review the structure and goals of the program, and assess the participants’ current life situation and PA status using a standardized questionnaire [87]. Participants’ previous PA experiences and preferences are also explored. To identify individual motives and goals related to PA, the Bernese Motive and Goal Inventory [88] is administered.

In the second session, these motives and goals are reviewed and reflected upon to derive personal implications for PA engagement. A first structured PA session is identified, and relevant logistical and motivational conditions for attendance are established. The third session involves attending this first exercise session together with the coach providing on-site support. During this and subsequent sessions, affective responses are monitored using the Feeling Scale [89], the Felt Arousal Scale [90] is used to assess perceived activation, and the Borg Perceived Exertion Scale [91] is administered for the definition of exertion.

Sessions 4 to 9 alternate between attending structured PA sessions and reflective sessions. These reflective coaching sessions involve evaluation of experiences, discussing whether specific activities should be continued or adapted, and planning future participation to optimize enjoyment and fit with individual preferences.

All 10 coaching sessions are conducted face-to-face in the participant’s personal environment to maximize individual support and real-world applicability. Each session is scheduled to last approximately 60 min.

To further facilitate behavior change, the coaching is supported by a wearable smart ring, adhering to 12-point WATCH (Wearable Activity Tracker Checklist for Healthcare) guidelines [92]. Specifically, the Oura Ring (Generation 3; Ōura Health Oy, Finland) is used. The lightweight device (4–6 g) includes a 50-Hz triaxial accelerometer, NTC thermistor, and photoplethysmograph to continuously measure movement, body temperature, heart rate, heart rate variability, and respiration [93]. The ring is worn on the nondominant hand (index, middle, or ring finger). The device comes with a 6–7-day battery life and uploads data to both a smartphone app and a secure cloud dashboard. This allows coaches to monitor participants’ PA levels, energy expenditure, and sleep patterns. Participants in IG1 continue to wear the ring after the end of the 10-week coaching program until follow-up.

### Intervention Group 2

Participants assigned to IG2 receive the same 10-week interest- and experience-based coaching as IG1 and also continue to wear the Oura Ring until follow-up. However, unlike IG1, IG2 remains in contact with the coach for an additional 6 months (26 weeks) to receive cognition-based coaching aimed at promoting sustainable behavior change.

During this phase, biweekly phone calls are conducted to assess participants’ PA habits and adherence to their routines, offering support if difficulties arise. These brief (~20-min) phone sessions focus on applying evidence-based behavior change techniques (BCTs) to reinforce long-term habit formation [94]. Strategies include structured activity planning, identifying and overcoming barriers, leveraging social support, and relapse management. The goal is to enhance self-regulation and resilience, ensuring that participants maintain their achieved activity levels beyond the initial intervention period. A detailed description of the intervention procedures is provided in the Intervention Guide, which is available as Additional file 1.

### Dose rationale for intervention groups

Exercise guidance in this trial is informed by the World Health Organization’s recommendations, which advise a minimum of 150 min of moderate-intensity or 75 min of vigorous-intensity aerobic PA per week or an equivalent combination of both [95]. However, these targets may not be attainable for all individuals with MDD, and, conversely, some participants may already meet them at baseline [60]. Therefore, the intervention emphasizes an individualized, adaptable approach. Recent evidence indicates that as little as 50 min of moderate-intensity aerobic exercise per week can reduce depressive symptoms, with optimal effects observed at approximately 140 min weekly [96]. Participants are encouraged to increase their activity at any level, highlighting the benefits of even light-intensity movement [97, 98]. PA recommendations are framed as aspirational and adaptable, aligning with current efforts to reframe PA guidelines in a more effective manner [99, 100]. A gradual, stepwise progression toward an active lifestyle is promoted, emphasizing enjoyment, feasibility, and sustainability. To support individual preferences and enhance engagement, a range of activity types is encouraged, including aerobic and resistance training, yoga, and individual and team sports.

### Control group

The control group (CG) receives treatment as usual provided by the day clinic, along with standardized written information on the health benefits of PA. Specifically, they are sent a 62-page booklet titled *Bewegungsempfehlungen Schweiz: Grundlagen*, published in 2022 by the Swiss Federal Office of Sports in collaboration with other members of the “hepa” network [81].

### Criteria for discontinuing or modifying allocated interventions {11b}

Participants can withdraw from the study at any time without providing a reason and without any impact on their medical care. Participants are asked to discontinue from the study if they develop any cardiovascular, metabolic, neurological, pulmonary, or orthopedic complications that could limit the safety of participation in PA. Further, female participants who become pregnant during the course of the project are excluded due to the considerable influence of pregnancy on PA behavior [101, 102].

### Strategies to improve adherence to the interventions {11c}

Adherence of participants to the two intervention conditions is enhanced through regular contact with their personal PA coach. Additionally, the smart ring and accompanying mobile app facilitate self-monitoring of PA behavior and thereby promote daily engagement with health-related metrics. Monitoring of adherence to the intervention conditions is achieved through documentation of smart ring data and coaching session contents by the PA coaches.

Participants of all three groups (CG, IG1, and IG2) receive written feedback on cardiorespiratory fitness, PA level, and body composition after each data assessment (T0, T1, T2). This feedback includes graphical visualization with comparison over time and may increase self-awareness, perceived progress, and motivation.

### Relevant concomitant care permitted or prohibited during the trial {11d}

All study participants follow treatment as usual in the day clinic. Apart from this, there are no requirements or restrictions on concomitant other treatments.

### Provisions for posttrial care {30}

In the event of study-related damage or injuries, the liability of the University of Basel, Medical Faculty, provides compensation, except for claims that arise from misconduct or gross negligence.

## Outcomes {12}

### Primary outcome

#### Device-based PA

The primary outcome is the change in device-measured PA over time, measured using a Fibion® SENS device (Fibion Inc., Jyväskylä, Finland) over 7 consecutive days at the three timepoints. The device is attached to the anterior thigh (centerline, one-third from the distal end) and sealed in a waterproof covering with medical adhesive tape. Participants wear it continuously, 24 h/day, facilitated by its low-maintenance design (no charging required). The sensor records raw triaxial acceleration at 12.5 Hz and classifies PA type (sitting, long sitting, standing, walking, cycling) and intensity (light [LPA], moderate [MPA], vigorous [VPA]) with high accuracy [103]. Reliability and validity have been demonstrated in previous research [104–109]. To enhance data accuracy, participants record their bedtime, ensuring that sleep periods are excluded from analysis to prevent an overestimation of sedentary time. At the end of the 7-day period, participants remove adhesive tape and return the Fibion® SENS device at the day clinic.

### Secondary outcomes

#### Self-reported physical activity

The 7-Day Physical Activity Recall (7DPAR) is used to assess participants’ self-reported PA through a structured 20-min interview by a trained research assistant [110–112]. The 7DPAR assesses all PA bouts of at least 10-min duration over the past 7 days. For a PA bout to be recorded, intensity needs to be at least moderate [112]. Interviewers record breaks during PA, which are subtracted from total durations. In addition to total minutes of PA in moderate, moderate to vigorous, and vigorous intensity per week, the 7DPAR also captures strength and flexibility training. As part of this study, the 7DPAR is supplemented by a question on participation in structured exercise and sports programs. This latter component is particularly relevant, as the coaching intervention aims to support engagement in such programs [113].

#### Depressive symptom severity

Depression severity is assessed using the Hamilton Depression Rating Scale (HAMD-17) [114] and the self-report Beck Depression Inventory-II (BDI-II) [115, 116]. The HAMD-17 is administered as a structured clinical interview by trained study personnel. The BDI-II is a 21-item questionnaire that assesses affective, behavioral, cognitive, and somatic symptoms of unipolar depression. Total scores range from 0 to 52 for the HAMD-17 and from 0 to 63 for the BDI-II, with higher scores indicating greater depression severity. Both instruments demonstrate acceptable reliability and validity [117–119]. Treatment response is defined as a ≥ 50% reduction in HAMD-17 score from baseline to endpoint, partial response as a 25%–49% reduction, and nonresponse as a < 25% reduction. Remission is defined as a HAMD score ≤ 7 [120]. For the BDI-II, response is similarly defined as a ≥ 50% reduction and remission as a score ≤ 9; no consensus definitions exist for partial response [121]. According to the German S3 clinical guidelines [122], HAMD-17 scores ≥ 17 indicate at least moderate depression, whereas a BDI-II score of ≥ 20 reflects moderate symptom severity [123, 124].

#### Health-related quality of life

The Medical Outcomes Study 12-Item Short-Form Health Survey (SF-12) is used to assess participants’ self-perceived health [125]. As one of the most widely applied instruments in population health research [126], the SF-12 has well-documented reliability and validity [127, 128]. Composite scores for physical and psychological health are calculated according to the SF-12 scoring manual, with higher scores indicating better health functioning. To our knowledge, no minimal clinically important difference (MCID) data specific to patients with MDD are currently available; therefore, we reference MCID values identified in patients with low back pain, where improvements of > 3.77 points in the psychological subscale and > 3.29 points in the physical subscale have been established [129].

#### Social-cognitive and affective determinants

To evaluate the effectiveness of the theory-based coaching intervention in influencing the targeted psychological mechanisms, a range of social-cognitive (reflective) and affective determinants are assessed.

##### Exercise-related self-efficacy

Exercise-related self-efficacy is assessed using three items measuring confidence in initiating, maintaining, and resuming exercise after a relapse (e.g., “I feel confident to start a new exercise activity”). This scale has demonstrated acceptable psychometric properties [130] and has been used in previous intervention studies [131–133]. Responses range from 0 (not at all confident) to 5 (completely confident), with the total score calculated as the sum of all three items. There are currently no established MCID scores for this index.

##### Exercise-related outcome expectations

Outcome expectancies are assessed using nine positively framed (“pros”) (e.g., “Regular exercise improves my physical appearance”) and seven negatively framed (“cons”) items (e.g., “Exercise puts me in situations where I feel embarrassed”) [134]. Items are rated on a 4-point Likert scale from 1 (not true) to 4 (completely true). The scale has demonstrated satisfactory psychometric properties [134]. Composite scores for positive and negative expectancies are calculated as the arithmetic means of their respective items. There are currently no established MCID scores for this index.

##### Exercise intention

Exercise-related goal intentions are assessed using a single item measuring the strength of participants’ intention to exercise regularly over the coming weeks and months (0 = no intention, 5 = very strong intention) [130]. This measure has demonstrated acceptable reliability and validity in previous studies [135, 136]. There are currently no established MCID scores for this index.

##### Exercise-related self-concordance

Exercise-related self-concordance is assessed using the 12-item SSK scale [135], based on the self-concordance model by Sheldon and Elliot [137]. The scale comprises four subscales measuring intrinsic (e.g., “I exercise because it’s fun for me”), identified (e.g., “I exercise because I have good reasons to be physically active”), introjected (e.g., “I exercise because otherwise I would feel guilty”), and extrinsic (e.g., “I exercise because others tell me to”) motivations for exercising. Items are rated on a 6-point Likert scale (1 = not at all true, 6 = completely true). An overall self-concordance index is calculated by summing the intrinsic and identified mean scores and subtracting the introjected and extrinsic mean scores. The SSK scale has demonstrated satisfactory reliability and validity [135]. There are currently no established MCID scores for this index.

##### Exercise-related action planning

Action planning is assessed using a 5-item index with established reliability and validity [137, 138], measuring the extent to which participants have pre-planned their exercise participation. Specifically, participants are asked about their planning regarding when, where, how, how often, and with whom they exercise. Responses are rated on a 4-point scale (1 = not at all true, 4 = completely true), and item scores are summed to create an overall index. There are currently no established MCID scores for this index.

##### Perceived barriers to exercise

Perceived exercise-related barriers are assessed using a 19-item scale that identifies various obstacles to regular exercise participation [139]. The instrument has demonstrated satisfactory psychometric properties [54, 139]. Participants rate how often they perceive each barrier (e.g., “I have too much work to do”) on a 4-point Likert scale (1 = almost never, 4 = almost always). The mean score is calculated to provide a single overall score. There are currently no established MCID scores for this index.

##### Exercise-related coping planning

Exercise-related coping planning is assessed using a 5-item index [138]. Participants are asked to what extent they use self-regulation strategies to overcome potential barriers to exercise (e.g., “I have made a detailed plan for what to do in difficult situations to act according to my intentions”). Responses are rated on a 4-point scale (1 = not at all true, 4 = completely true). Item scores are summed to create a composite index. The scale’s reliability and validity have been established in previous research [136, 138]. There are currently no established MCID scores for this index.

##### Exercise-related social support

Social support for exercise is assessed using a 7-item index [134], focusing on support from relevant others (e.g., “Close family or friends help me plan my exercise”). Responses are given on a 4-point Likert scale (1 = almost never, 4 = almost always). Item scores are summed to create a composite index. The scale has demonstrated psychometric soundness in previous studies [133, 134]. There are currently no established MCID scores for this index.

##### Explicit affective attitudes towards exercise

Participants’ affective evaluations of past exercise experiences are assessed using the Affective Exercise Experiences questionnaire (AFFEXX), a 36-item instrument developed to capture explicit affective attitudes toward exercise [66]. The AFFEXX includes 10 subscales, organized into 3 overarching domains: (1) antecedent cognitive appraisals, (2) core affective exercise experiences, and (3) attraction vs. antipathy as a motivational outcome. The antecedent cognitive appraisal domain comprises six subscales that assess constructs such as competence-incompetence, interest-boredom, and pride/honor-shame/guilt. These cognitive appraisals are theorized to shape the core affective exercise experience, which includes three subscales: pleasure-displeasure, energy-tiredness, and calmness-tension. In turn, these affective responses are assumed to influence the motivational outcome, assessed via a single subscale capturing the degree to which exercise is experienced as attractive or aversive. Each of the 36 items presents a pair of bipolar statements, and participants rate the extent to which their views and experiences align with either pole using a 7-point Likert scale (1 = perfectly matching left statement, 7 = perfectly matching right statement). Scoring is performed using the official scoring script provided by the questionnaire developers [140]. Reverse-coded items are recoded before calculating subscale scores. Each of the 10 subscales is scored independently. In addition, composite scores are calculated for the domains of antecedent cognitive appraisals (sum of six subscales) and core affective experiences (sum of three subscales). The validity of this instrument has been documented in previous research [66, 141]. The MCID for this instrument has not been determined yet.

##### Implicit attitudes towards exercise

Automatic affective evaluations of exercise are assessed by using the Single Target Implicit Association Test (ST-IAT), administered via E-Prime 2.0 (Psychological Software Tools, USA). Unlike standard IATs, which compare associations between evaluative concepts (e.g., “good” vs. “bad” and “physical activity” vs. “physical inactivity”), the ST-IAT assesses attitudes toward a single target concept — exercise — without a direct comparison. The ST-IAT employed in this study has shown reliability and discriminant validity [142]. In this version, “exercise” serves as the target concept, while “good” and “bad” are the evaluative categories. The target stimuli are photographs of middle-aged adults engaging in exercise activities (e.g., swimming, running), and these images show individuals without emotional expressions (e.g., no smiling or pained faces). Rather than using attribute words, emoticons (eight smileys and eight frownies) are used, which reduces potential confounds related to verbal abilities and ensures visual stimulus consistency [143]. The task consists of 2 phases: The first phase includes 16 practice trials, where participants categorize emoticons as either “good” or “bad.” The second phase consists of two blocks, where participants assign emoticons and target images to the “exercise-good” or “exercise-bad” categories. The order of the blocks is counterbalanced across participants, and after the first block, the assignment of exercise to the evaluative categories is reversed. Each block includes 32 trials, preceded by 16 practice trials to minimize learning effects. Inaccurate responses are repeated until a correct response is recorded. Response speed and accuracy are emphasized, with participants responding using their left or right index finger. The primary dependent variable is the D-score, calculated by dividing the difference in mean reaction time between the two blocks by the within-individual standard deviation of reaction times across compatible and incompatible trials [144]. The D-score ranges from −2 to +2, with |0.15|, |0.35|, and |0.64| indicating slight, moderate, and strong preference (or aversion), respectively [145].

##### Physical self-perception

Physical self-perception is assessed using the Physical Self-Description Questionnaire (PSDQ), a self-report tool designed to evaluate various dimensions of physical self-concept [146]. In this study, we focus on 4 subscales: endurance, flexibility, coordination, and strength, comprising a total of 14 items. Each item presents a statement that participants rate according to their level of agreement or disagreement. Subscale scores are calculated by averaging the responses to the items within each subscale, with higher scores indicating more positive self-perceptions in the respective areas. Extensive psychometric evaluation of the PSDQ indicates that it is a reliable and valid instrument [147–149].

### Potential moderators

#### Body mass index, percentage of body fat, and waist circumference

Body weight is measured using a digital scale (BC-545, Tanita, USA), without shoes and to the nearest 0.1 kg, with participants in light clothing. Height is measured using a stadiometer, with participants standing barefoot, to the nearest 0.5 cm. BMI is calculated using the formula: weight (kg)/height^2^ (m^2^). According to WHO standards [150], participants are classified as overweight with a BMI ≥ 25.0 kg/m^2^ and obese with a BMI ≥ 30.0 kg/m^2^. A 5% weight loss is considered as the MCID for those classified as overweight or obese [151]. The BC-545 scale also performs bioelectrical impedance analysis, measuring body fat percentage. For healthy body fat, the WHO [150] recommends a maximum of 32% for women and 25% for men. A 2% reduction in body fat percentage is defined as the MCID [152]. Waist circumference is measured using a flexible tape at the natural waist (midway between the ribcage and iliac crest). According to the National Cholesterol Education Program [153]^153^, a waist circumference ≥ 80 cm for women and ≥ 94 cm for men turned out to be a risk factor for metabolic syndrome.

#### Cardiorespiratory fitness

Cardiorespiratory fitness is considered a potential moderator to determine whether the effectiveness of the intervention is influenced by participants’ initial fitness levels. This is supported by previous research indicating that the effects of an affect-based exercise prescription were moderated by cardiorespiratory fitness levels [154]. To assess cardiorespiratory fitness, we use the Åstrand indirect test of maximal oxygen uptake (VO_2_max) [155], performed on a bicycle ergometer (Ergoselect 200), which is validated for measuring submaximal fitness [155]. Gender- and age-adjusted cutoffs categorize participants into low, moderate, and high fitness groups [155]. The reliability and validity of the Åstrand nomogram and its linear extrapolation for deriving VO_2_max have been well documented [156]. A submaximal fitness test is employed, as more than half of individuals with depression do not meet the primary or secondary criteria for maximal exertion in cardiopulmonary exercise testing [157].

#### Medical history

Psychiatric medical history is assessed as a potential moderator. Using a structured questionnaire, participants provide information on current and past depressive episodes, treatments—including medication use—and comorbid diagnoses.

#### Sociodemographic background

Sociodemographic background is assessed through a structured questionnaire. Participants provide information on their age, mother tongue, nationality, relationship status, educational background, work experience, the presence of children in the household, smoking status, and household income.

### Implementation evaluation

#### Reach, dose, fidelity, and adaptation

To comprehensively assess the intervention’s effectiveness, its implementation is systematically analyzed. This evaluation follows the framework proposed by the Medical Research Council (MRC) [158], which has been previously applied for the implementation evaluation of a PA counselling in in-patients with MDD [159] to assess implementation processes. Specifically, four key components are examined: (1) Reach—assessing whether the intervention successfully engages the intended target group; (2) dose—evaluating the duration and number of administered intervention sessions; (3) fidelity—determining adherence to the prescribed administration protocol; and (4) adaptation—identifying any modifications made to the intervention protocol.

The above-described analyses are based on quantitative data collected from coaches. Thus, coaches document key aspects of their sessions using a standardized tool. This documentation includes session dates, preparation time, coaching duration, follow-up time, the Feeling Scale (FS) [89], Felt Arousal Scale (FAS) [90] and Borg Perceived Exertion Scale (Borg) [91], and session content, e.g., BCTs employed.

#### Participant satisfaction

Additionally, participant satisfaction data are gathered via questionnaires administered at post-intervention (T1: IG1 and IG2) and follow-up (T2: IG2). The questionnaire, adapted from a previous project [159], assesses participants’ expectations, comprehension of coaching content, satisfaction with the coach, motivation to continue, likelihood of recommending the intervention, perceived suitability for promoting PA, overall satisfaction, and perceived effort in relation to outcomes. Responses are recorded on a 4-point Likert scale (ranging from “no” to “yes”), while perceived effort is assessed using a scale from “too high” to “low.” Additionally, participants report their perceptions of coaching session intervals, with response options ranging from “too long” to “too short.”

### Participant timeline {13}

Patients are screened within their first week of admission to the day clinic and invited to participate in the study. Interested individuals receive detailed study information from designated study personnel. Recruitment continues until 114 participants have been enrolled. Following informed consent, baseline assessments are initiated promptly. As the 10-week intervention is designed to run concurrently with the 12-week standard treatment in the day clinic, timely initiation of baseline assessments is ensured. The participant timeline is illustrated schematically in Table 1.

**Table 1:**
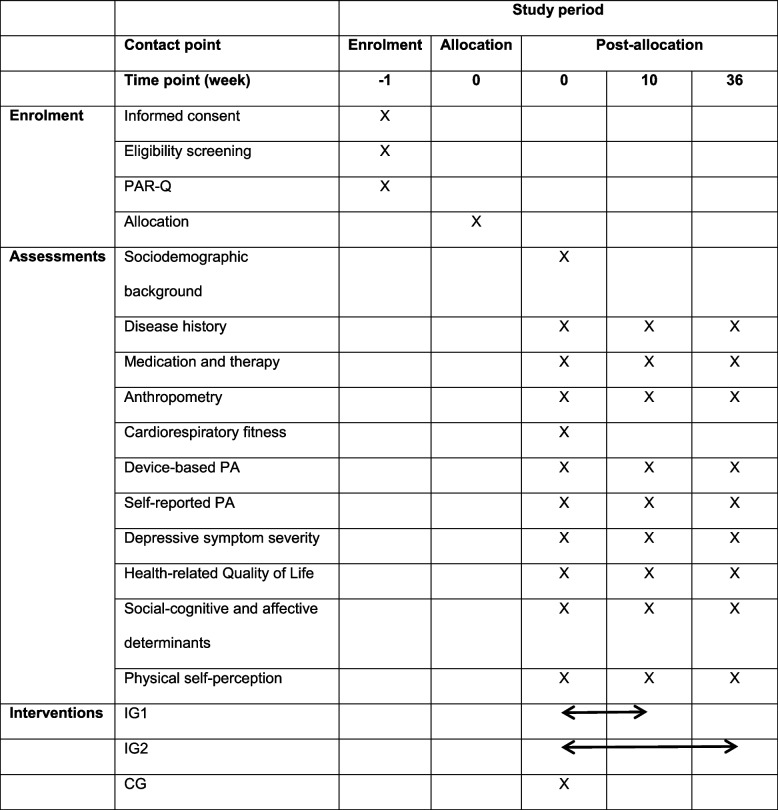
Schematic illustration of participant timeline in accordance with SPIRIT guidelines

### Sample size {14}

Based on the expectation of a weak-to-moderate effect size (*f* = 0.175) for the primary outcome of change in device-based PA, an alpha error probability of 0.05, and a correlation between repeated measures of *r* = 0.50, 29 participants per group (87 in total) are required to achieve 90% power via ANOVA (G*Power 3.1.9.7). The assumed effect size was informed by previously published comparable RCTs [160, 161]. The final sample size is increased by 30% (*N* = 114) to account for anticipated attrition, defined as loss to follow-up with missing primary outcome data. This adjustment aligns with a previously published RCT involving in-patients receiving PA counselling, which reported a dropout rate of 30% [162].

### Recruitment {15}

Participants are recruited from two day clinic groups, which collectively treat 56 patients at any given time, each undergoing a 12-week treatment program. This corresponds to an average screening rate of five patients per week. Assuming the majority of patients meet the eligibility criteria, an average enrollment rate of 1.5 participants per week is anticipated, resulting in a projected recruitment period of 1.5 years. If necessary, the recruitment period may be extended to a maximum of 2 years.

### Assignment of interventions: allocation

#### Sequence generation {16a}

Participants are randomly allocated in a 1:1:1 ratio to one of two PA coaching conditions (with or without continuous support after post-intervention) or to a control condition receiving general information about health-enhancing PA. The allocation sequence was computer-generated using the Sealed Envelope^™^ randomization service (Sealed Envelope Ltd., London, UK), employing random permuted blocks of 3 and 6. Randomization is stratified by age (< 35 years vs. ≥ 35 years) and sex (male vs. female) to ensure balanced distribution of these variables across the three study arms. The randomization list was prepared by an independent researcher not otherwise involved in participant recruitment, enrollment, or assessment.

#### Concealment mechanism {16b}

After the randomization list was generated, a staff member employed at DSBG who has no further role in the study except data monitoring used it to prepare sealed envelopes with treatment assignments, ensuring allocation concealment.

#### Implementation {16c}

After completing the baseline assessment, each participant draws a sealed envelope to determine their allocation. The research assistant who conducts the assessments then informs the participants of their assigned condition.

### Assignment of interventions: blinding

#### Who will be blinded {17a}

Because of the behavioral nature of the intervention, participants and researchers cannot be blinded.

#### Procedure for unblinding if needed {17b}

This is not applicable, as the study is open-label.

## Data collection and management

### Plans for assessment and collection of outcomes {18a}

All outcome measures are systematically recorded on-site at the day clinic across the three measurement timepoints. To ensure methodological consistency and data integrity, all study personnel have undergone rigorous training and adhere to standardized operating procedures. Participant responses to the questionnaires are captured electronically via REDCap to minimize transcription errors and enhance data accuracy. The rationale underpinning the selection of specific measurements, as well as their reliability and validity, is described under the " Outcomes {12}" section.

### Plans to promote participant retention and complete follow-up {18b}

In week 26 of the 36-week study period, corresponding to 16 weeks post-completion of the day clinic treatment, participants in all three groups receive a small token of appreciation by mail from the study team. The token consists of a thank-you postcard, a small motivational sticker, and a piece of chocolate. All items are non-monetary and of minimal value and are intended to acknowledge participants’ contributions to the study and to promote sustained engagement in the completion of follow-up study procedures.

For participants allocated to the intervention groups, upcoming data assessments are integrated into ongoing coaching sessions as part of the established protocol. In contrast, participants in the control group are notified of forthcoming assessments via written communication.

Importantly, individuals who discontinue participation in the intervention are still invited to complete scheduled data assessments, thereby supporting the integrity and completeness of longitudinal data collection.

### Data management {19}

Data are entered into electronic case report forms (eCRFs) using REDCap. Questionnaires are entered directly by the participants through the survey function. All other assessments are recorded on paper CRFs and manually transferred to REDCap by trained study personnel.

### Confidentiality {27}

Participant confidentiality is safeguarded through pseudonymization using a unique four-digit ID. Personal identifiers are stored separately in an encrypted, access-restricted file. Only authorized personnel have access to identifiable data, and all study records are handled in compliance with data protection regulations. Anonymized data are used for analysis, publication, and data sharing.

### Plans for collection, laboratory evaluation, and storage of biological specimens for genetic or molecular analysis in this trial/future use {33}

This is not applicable, as no biological specimens are collected.

## Statistical methods

### Statistical methods for primary and secondary outcomes {20a}

Descriptive statistics are presented as means (M), standard deviations (SD), medians (Mdn), interquartile ranges (IQR), frequencies, and percentages (%). The primary outcome, device-based PA (Fibion^®^), and the secondary outcomes, depressive symptom severity (HAMD17, BDI-II) and health-related quality of life (SF-12), will be analyzed using linear mixed models (LMMs) with measurements at T1 and T2 as outcomes. Baseline values of the respective outcomes (T0) will be included as predictors, along with a baseline × time interaction to account for potential changes in baseline influence over time. Fixed effects will include group (CG, IG1, IG2), time (T1, T2), and the group × time interaction, with a random intercept for each participant. For the secondary outcomes, device-based PA at the corresponding time point will additionally be included as a predictor to examine whether changes in these outcomes are associated with PA. Estimated marginal means from the LMMs will be used to compare groups at each time point, with Dunnett’s adjustment applied for comparisons of each intervention group versus control.

Although randomization is expected to minimize baseline imbalance, any clinically relevant imbalances observed will be accounted for in adjusted analyses of the primary outcome.

Continuous predictors (e.g., age, BMI, baseline PA) will be examined for potential nonlinear effects and may be modeled using flexible approaches such as natural splines. Model assumptions, including normality of residuals and homoscedasticity, will be checked, and appropriate transformations or alternative methods will be applied if assumptions are seriously violated.

Baseline CRF will be tested as a moderator by including CRF and its interactions with group and time (CRF × group × time) in the LMMs. Models will first be run without CRF to estimate the overall intervention effect.

Implementation outcomes (reach, dose, fidelity, adaptation) and participant satisfaction will be summarized descriptively. Between-group differences (IG1 vs. IG2) will be explored using independent-sample *t*-tests. All statistical analyses are performed using SPSS and Jamovi [163], with the significance level set at *p* $\le$ 0.05. The completed trial will be reported in accordance with the CONSORT 2025 statement [164].

### Interim analyses {21b}

No interim analyses are planned. All planned analyses (e.g., pre- to post-intervention effects, follow-up comparisons) are conducted after completion of data collection for the respective time points.

### Methods for additional analyses (e.g., subgroup analyses) {20b}

No additional or subgroup analyses are planned beyond those described for the primary and secondary outcomes.

### Methods in analysis to handle protocol non-adherence and any statistical methods to handle missing data {20c}

All study variables will be screened for missing data, outliers, and multicollinearity. Missing data patterns will be examined descriptively and visually, with Little’s MCAR test as a supplementary tool. The most plausible missing data mechanism (MCAR, MAR, or MNAR) will be inferred from these observations, study design, and theoretical considerations, and sensitivity analyses will be conducted to assess robustness [165]. If data are plausibly MCAR or MAR, appropriate imputation methods (e.g., multiple imputation) will be applied [166].

The primary analysis will follow the intention-to-treat (ITT) principle, including all participants as randomized and using the chosen imputation methods for missing data [166]. This approach estimates the effectiveness of the interventions under real-world conditions [167, 168]. A secondary per-protocol (PP) analysis, including only participants who adhered to the study protocol, will be conducted to estimate the intervention effect under ideal adherence [167].

### Plans to give access to the full protocol, participant-level data, and statistical code {31c}

The full study protocol can be made available upon reasonable request to the study sponsor. Access to the participant-level dataset and statistical code is not granted, as participants are not asked to provide consent for broader data sharing beyond the scope of the PACOUTPAT study. According to the terms outlined in the informed consent form, data may not be used for scientific purposes other than those explicitly stated in the approved study protocol.

## Oversight and monitoring

### Composition of the coordinating center and trial steering committee {5d}

PACOUTPAT is conducted as a single-center trial at the Sonnenhalde AG Psychiatric Clinic. The study is implemented by the Department of Sport, Exercise and Health (DSBG) of the University of Basel at the Sonnenhalde AG site.

The sponsor-investigator (MG) from the University of Basel is responsible for the overall coordination and quality assurance of the trial, including implementation in accordance with the protocol, data management and analysis, staff instruction, and dissemination of findings. Day-to-day trial operations are led by an affiliated doctoral student (CS) who oversees participant recruitment, data assessments, and intervention delivery. The doctoral student is supported by a research assistant who assists with recruitment, data collection, and communication with patients. PA coaches, all with a background in sport and exercise science, are trained by the doctoral student and deliver the intervention according to standardized procedures. The co-principal investigator (J. B., chief physician of Sonnenhalde AG) is responsible for the implementation of clinical treatment and the supervision of patients. Furthermore, he ensures favorable recruitment conditions by integrating the intervention into treatment as usual and establishing effective communication between clinic staff and DSBG personnel. Clinic staff support recruitment by informing eligible patients about the study and encouraging contact with the research team.

The study team maintains structured communication through the following:

- Weekly to monthly meetings between the sponsor-investigator and the doctoral student
- Weekly to biweekly meetings between the doctoral student and the research assistant
- Weekly to monthly meetings between the doctoral student or research assistant and the PA coaches
- Weekly meetings between the doctoral student or research assistant and the clinic staff

Additionally, an annual team meeting is held with the broader research team, including the sponsor-investigator, co-principal investigator, doctoral student, research assistant, PA coaches, and co-authors, to review study progress and coordinate next steps.

### Composition of the data monitoring committee and its role and reporting structure {21a}

The Department of Sport, Exercise and Health (DSBG) is fulfilling the monitoring duties associated with the present trial. Data monitoring is overseen by the secretary of the Psychosocial Health and Physical Activity Research Unit at the DSBG. A monitoring visit at 12 months and a close-out visit are performed according to the monitoring plan. Electronic files (and their backups) and other documents containing participant data are kept accessible and monitored. The sponsor answers all questions that arise from the monitoring procedures.

### Adverse event reporting and harms {22}

Adverse events are systematically recorded at both post-intervention and follow-up assessments. In the event of a serious adverse event, documentation is completed and reported to the study sponsor within 24 h. Additionally, PA coaches monitor participants throughout the intervention period and report any adverse events directly to the research team for initiation of documentation.

### Frequency and plans for auditing trial conduct {23}

The trial is under the oversight of the Ethics Committee of North-Western Switzerland (EKNZ; project ID 2025-00557). Formal auditing procedures are not planned but can spontaneously be announced and performed by the ethics committee. Any modifications to the study protocol (amendments) or occurrence of severe adverse events are reported to the ethics committee by the research team.

### Plans for communicating important protocol amendments to relevant parties (e.g., trial participants and ethical committees) {25}

Substantial changes to the study setup and study organization, the protocol, and relevant study documents are submitted to the ethics committee for approval before implementation. Under emergency circumstances, deviations from the protocol to protect the rights, safety, and well-being of participants may proceed without prior approval of the ethics committee. Such deviations shall be documented and reported to the ethics committee as soon as possible. Non-urgent changes are only implemented following formal approval.

### Dissemination plans {31a}

Following the processing and analyses of collected data, manuscripts containing the findings of the study are planned to be published within 12 months. For the purpose of publication in the public register, the sponsor also ensures that a lay summary of the trial results is entered in BASEC within 1 year of completion or discontinuation of the trial. The entry is made at least in the national languages of Switzerland in which the study participants were recruited.

## Discussion

Despite strong evidence supporting PA as an effective treatment for MDD [35–37], only a minority of individuals with MDD engage in sufficient levels of PA [51]. PA coaching has emerged as a promising intervention strategy and has demonstrated good acceptance among patients in previous work [61]. However, long-term behavior change remains a major challenge [162]. Recent advances in PA behavior change underscore the importance of addressing not only cognitive determinants but also affective processes [64, 65, 72]. Building on the new behavior change advances and the experience of previous research with in-patients [162], the present study introduces a novel PA coaching program tailored to outpatients with MDD. By explicitly fostering positive affective responses to exercise while also addressing cognitive-reflective factors, the intervention seeks to achieve sustainable increases in PA and, thereby, improve mental and physical health as well as overall quality of life. Behavior change is further supported through smart-ring wearable technology, which promotes self-monitoring and real-time feedback. To our knowledge, this is the first PA coaching intervention for individuals with MDD specifically that targets positive affective responses to exercise while simultaneously incorporating smart-ring technology to enhance PA engagement and adherence.

To contextualize the PACOUTPAT approach, findings from a recent personalized PA intervention for outpatients with MDD are considered, as the trial shares several key features with the current intervention and provides relevant insights. Keller-Varady et al. (2023) evaluated a 6-week personalized PA program supported by motivational interviewing (MI) in outpatients with MDD [169]. Like the current trial, their intervention was delivered in person, focused on establishing sustainable PA routines, and used coaching techniques to facilitate behavior change. The intervention group showed a successful increase in moderate-to-vigorous PA, which was maintained at the 3-month follow-up. However, the relatively small sample size raises concerns about the statistical power and generalizability of the findings. Participant feedback emphasized the importance of free access to personalized coaching, structured guidance in PA planning, and the availability of PA options close to patients’ homes. These insights align with core elements of the PACOUTPAT intervention and are likely to enhance its real-world feasibility.

This trial has several important strengths. Its pragmatic, real-world design is embedded within standard day clinic care, enhancing the potential for future implementation. The intervention is built on a solid theoretical foundation guided by the ART. It also builds upon earlier PA coaching research, integrating key learnings and advancing the field by addressing affective processes as critical drivers of sustained PA engagement. Lastly, smart-ring technology provides objective feedback that may strengthen adherence.

Taken together, the findings from this trial may offer valuable insights into PA promotion in individuals with MDD. Embedding affective-reflective PA coaching within routine psychiatric care could serve as a feasible and scalable strategy to counter physical inactivity. In doing so, the intervention holds promise to enhance both mental and physical health outcomes and support long-term lifestyle change in individuals with MDD.

### Trial status

The trial was prospectively registered in the DRKS registry for controlled trials on May 8, 2025 (DRKS00036209), protocol version 2. Recruitment began in June 2025 and is expected to be completed by December 2026. Data collection commenced in June 2025 and is scheduled to conclude in September 2027. For a brief overview of the study timeline and milestones, see additional file 2. This study follows protocol version 2, dated April 25, 2025 (EKNZ; project ID 2025-00557).

## Supplementary Information

Additional file 1: Intervention Guide.

Additional file 2: Timeline of PACOUTPAT-trial.

Additional file 3: SPIRIT 2025 checklist of items to address in a randomized trial protocol.

## Data Availability

Access to the final, de-identified trial dataset is restricted to the sponsor-investigator (MG) and the affiliated doctoral student (CS) at the Department of Sport, Exercise and Health, University of Basel. No external researchers or third parties have access to the dataset. This restriction is based on the terms outlined in the informed consent form, which specifies that data collected during the PACOUTPAT study may only be used for the purposes stated in the approved study protocol. Consequently, there are no provisions for data sharing beyond the core research team, and no contractual agreements permit broader access. Requests for access to anonymized data for secondary analyses are not granted, in adherence to the ethical commitments made to participants.

## Abbreviations

7DPAR: 7-Day Physical Activity Recall
AFFEXX: Affective Exercise Experiences
ANOVA: Analysis of variance
ART: Affective-Reflective Theory of physical inactivity and exercise
BCT(s): Behavior change technique(s)
BDI-II: Beck Depression Inventory II
BMI: Body mass index
CG: Control group
CRFs: Case report files
DALYs: Disability-adjusted life years
DSBG: Department of Sport, Exercise and Health, University of Basel
EKNZ: Ethikkommission Nordwestschweiz (Ethics Committee of Northwestern Switzerland)
F32: Depressive episode (ICD-10)
F33: Recurrent depressive disorder (ICD-10)
FAS: Felt Arousal Scale
FS: Feeling Scale
HAMD-17: Hamilton Depression Rating Scale
ICD-10: International Classification of Diseases, Tenth Revision
IG: Intervention group
ITT: Intention-to-treat
LMM: Linear mixed models
LPA: Light physical activity
MCAR: Missing completely at random
MCID: Minimal clinically important change
MDD: Major depressive disorder
MI: Motivational interviewing
MoVo: Motivation-volition
MPA: Moderate Physical Activity
MRC: Medical Research Council
MVPA: Moderate-to-vigorous physical activity
NTC: Negative temperature coefficient thermistor
PA: Physical activity
PACINPAT: Physical activity counselling in in-patients with major depressive disorder
PACOUTPAT: Physical activity coaching in outpatients with major depressive disorder
PAR-Q: Physical Activity Readiness Questionnaire
PP: Per protocol
PSDQ: Physical Self-Description Questionnaire
RCT: Randomized controlled trial
SF-12: Short Form-12 Health Survey
SSK-scale: Self-Concordance Scale
ST-IAT: Single-Target Implicit Attention Test
VO2max: Maximal oxygen uptake
VPA: Vigorous physical activity
WATCH: Wearable Activity Tracker Checklist for Healthcare
WHO: World Health Organization
