# Supplementary material for: Lifestyle physical activity coaching in outpatients with major depressive disorder (PACOUTPAT): study protocol for a randomized controlled trial on physical activity, depression, and quality of life
Source: Trials. 2026 Feb 17;27:231. doi: 10.1186/s13063-026-09500-1 (PMC13014798; doi:10.1186/s13063-026-09500-1)
Supplement: Supplementary file 1 — Additional file 1: Intervention Guide. [file 13063_2026_9500_MOESM1_ESM.pdf]

University of Basel
Department of
Sport, Exercise and Health

PACOUTPAT

Intervention Guide

Lifestyle Physical Activity Coaching in Outpatients with Major Depressive Disorders (PACOUTPAT):
Randomized Controlled Trial on Physical Activity, Depression and Quality of Life

Table of Contents

1 Overview 2
2 Interest- and experience-based coaching 3
Coaching 1 : Getting to know each other 3
Coaching 2 : BMZI and planning 9
Coaching 3 : Attendance of exercise session 11
Coaching 4 : Reflection on exercise programmes 13
Coaching 8 : Note on the study; reflection or visit of exercise session 16
Coaching 10 : Conclusion 20
3 Cognition-based follow-up coachings 23
Coaching 11: Setting goals 23
Coaching 12-16 und 18-21: Individual use of BCTs 26
Coaching 17: Interim status 28
Coaching 22: Preparation of end of coaching 30
Coaching 23 : Last coaching - conclusion 32

1 Overview

Content	Timepoint	Place	CG	IG1	IG2
Attaching the Fibion sensor	Week -1	Day clinic (DC)	X	X	X
**Baseline measurement**	Week 0	DC	X	X	X
Physical activity guidelines hepa	Week 1	E-Mail	X		
**Coaching 1**<br>**Getting to know each other,** building relationships, identifying interests in physical activity	Week 1	Face-to-face		X	X
**Coaching 2**<br>Discuss BMZI, **plan** initial and, if applicable, further visits to exercise facilities	Week 2	Face-to-face		X	X
**Coaching 3**<br>**Attending** an exercise class	Week 3	Face-to-face		X	X
**Coaching 4 until coaching 9**<br>**3x participation** in an exercise programme and **3x reflection** and planning according to individual needs; if necessary, independent participation in an exercise programme	Week 4 to Week 9	Face-to-face		X	X
Attaching the Fibion sensor	Week 9	DC	X	X	X
**Coaching 10**<br>**Conclusion,** sustainability strategies, outlook IG2	Week 10	Face-to-face		X	X
**Post measurement**	Week 10	DC	X	X	X
**Coaching 11 until FU-coaching 22**<br>Self-monitoring and feedback, ensuring sustainability, reflection, increasing everyday activity	Week 12 to Week 34	Remote			X
**FU-coaching 23**<br>Completion of coaching	Week 36	Remote			X
Attaching the Fibion sensor	Week 36	Home	X	X	X
**Follow-up (FU) measurement**	Week 37	DC	X	X	X

2 Interest- and experience-based coaching

**Coaching 1 : Getting to know each other**	
**Aims:**	• Establish a basis of trust (getting to know each other, building a relationship)<br>• Define the framework conditions for cooperation<br>• Assess the individual's starting point (living circumstances, previous experience with exercise)<br>• Clarify expectations and motivation (BMZI, interest in exercise, openness to new things)
**Duration**	60min
**Title**	**Content**
**Preparation**	● Study the baseline measurements of the coachee (initial situation). Note down important points in the coaching documentation template.<br>● Write an email to arrange an appointment for the first coaching session. This could look something like this:<br>*Subject: Appointment for your first physical activity coaching session (PACOUTPAT study)*<br>*Dear Ms/Mr [surname],*<br>*Thank you for participating in the PACOUTPAT study! My name is [first name and surname] and I will be your physical activity coach for the next [10 or 36] weeks.*<br>*To kick off our collaboration, I would like to schedule an appointment for our first coaching session (approx. 60 minutes). This session will take place in your personal environment – for example, at the day clinic, in a nearby park or café, or at your home.*<br>*Suggested dates:*<br>*[Option 1: Date, time]*<br>*[Option 2: Date, time]*<br>*If none of the suggestions suit you, please let me know which times are possible for you. I would also be grateful if you could let me know where you would like to meet. If you prefer, you are also welcome to contact me on [telephone number].*<br>*I look forward to meeting you soon and working together on your exercise goals.*<br>*Kind regards*<br>*[First name and surname]*
**Conversation**	
Introduce yourself and ask the coachee to introduce themselves	● Introduce yourself briefly, e.g. with your name, background (sports scientist), and perhaps something else that defines you.<br>● Ask the coachee to introduce themselves.<br>● Ask about motivation for participation and gather expectations.

	Example:<br>*I am pleased to meet you. My name is ... and I am a sports scientist. Would you mind if we addressed each other informally?*<br>• *How would you like to introduce yourself?*<br>• *What motivated you to participate in the study?*<br>• *What are your expectations regarding our collaboration?*
Clarify information about the procedure/framework conditions	Explain the personal health coaching process:<br>**Intervention group 1:**<br>*Over the next 10 weeks, we will conduct 10 coaching sessions of approximately 60 minutes each. There will be approximately one week between each coaching session. We will arrange the appointments so that they suit us both.*<br>• *Which days are usually best for you?*<br>• *What time is usually best for you?*<br>*We will meet in person in your personal environment. The aim of this is to enable us to plan and implement your new exercise habits directly in your environment. At the beginning, we will mainly talk and plan together. From the third coaching session onwards, we will also exercise together.*<br>*It is very important to me that we work together to find out which form of exercise you enjoy and which is good for you. The next step is to adapt your exercise routine and/or your everyday life so that you can continue with this form of exercise in the long term.*<br>**Intervention group 2:**<br>*Over the next 8 months, we will conduct 10 coaching sessions of approx. 60 minutes each, followed by 13 coaching sessions of 20 minutes each. In the first 10 weeks, there will only be about 1 week between coaching sessions. After that, there will be approx. 2 weeks between the shorter coaching sessions 11-23. We will arrange the appointments so that they suit us both.*<br>*During the first 10 weeks, we will meet in person in your personal environment. The aim of this is to enable us to plan and implement your new exercise habits directly in your environment. At the beginning, we will mainly talk and plan together. From the third coaching session onwards, we will also exercise together.*<br>*When the conversations become shorter from the 11th coaching session onwards, we will conduct them by telephone.*<br>*It is very important to me that we work together to find out which form of exercise you enjoy and which is good for you. The next step is to adapt your exercise routine and/or your everyday life so that you can continue this form of exercise in the long term.*
Communication channel	Agreement on communication channels<br>• Channel for scheduling appointments?<br>• Channel for communicating agreements?<br>• Intervention group 2: Channel for telephone conversations?<br>*‘If you are unable to attend an agreed appointment, please notify me (by text message or as otherwise agreed) as early as possible so that we can*

	reschedule.'
Basic idea of coaching	A good place to start is to ask whether the person already has any coaching experience and, if so, to what extent. This can be followed up by explaining the principles of coaching:<br>• *There is no one-size-fits-all solution: we take an individual approach and focus on personal resources.*<br>• The coach helps you to help yourself. The coach supports the coachee in finding their own solutions. The coachee is the expert on their own life situation.<br>• Misunderstandings and uncertainties should/may be addressed openly. *'It is important to me that you feel confident after completing the coaching and can implement the planned/discussed content independently.'*<br>• *'If we notice that we are not making progress' or 'the collaboration is being impaired because, for example, you do not like me, I ask you to address this openly so that we can find a solution together, as this should not be an obstacle to participation.'* If no joint solution can be found -> information about a possible change of coach.<br>Mention that we greatly appreciate punctuality and reliability for the agreed appointments. *'The conversations take place at the agreed time. We have to stick to this because I am coaching other people and have other tasks in my life. If you miss the appointment by a few minutes, you are welcome to join us later. Otherwise, we will have to make a new appointment.'*
Oura Ring and App	*The coaching is supported by the Oura Ring. You can see the measured values in the accompanying app. In preparation for our meetings, I will check the app to see how your week has been so that we are both on the same page. Please rest assured that I will not be evaluating your physical activity; this is simply part of my preparation.*<br>• *Do you have any questions about the ring and/or the app?*
Clarify open questions	Clarify questions/uncertainties
Check current living conditions	General living situation<br>• Current everyday life? Typical day?<br>• Professional/private commitments?<br>• *Who lives with you in your household?*<br>• *What else should I know about your everyday life? Any special circumstances?*<br>Health and well-being<br>• *How are you feeling physically and mentally at the moment?*<br>• *What else should I know about your state of health?*
Ask about previous and current physical activity (BSA questionnaire)<br>• The following questionnaire serves as a structure for an open discussion about previous and current exercise experiences.	

• At the same time, it can also be used to assess the current situation and thus be updated and compared at the end of the coaching session. When answering the questions, also refer to what the coachee has already told you.

Physical Activity and Sports Questionnaire (BSA)	1	Are you employed (including housewives/househusbands) or in training?

Yes – continue with Question 2	No – continue with Question 3

2	Your professional activity or training includes...

Sedentary Activities	none	Rather little/ somewhat	Rather more	A lot
Moderate Activity	none	Rather little/ somewhat	Rather more	A lot
Intense Activity	none	Rather little/ somewhat	Rather more	A lot

3	How many days and for how long did you engage in the following activities in the last 4 weeks?

Walking to work (including longer sections)	On ….. days during the 4 weeks	Appr.. ….. minutes per day	Not done
Walking to the shops	On ….. days during the 4 weeks	Appr.. ….. minutes per day	Not done
Cycling to work	On ….. days during the 4 weeks	Appr.. ….. minutes per day	Not done
Cycling for other purposes of transportation	On ….. days during the 4 weeks	Appr.. ….. minutes per day	Not done
Going for a walk	On ….. days during the 4 weeks	Appr.. ….. minutes per day	Not done
Gardening (e.g. mowing the lawn, trimming hedges)	On ….. days during the 4 weeks	Appr.. ….. minutes per day	Not done
Physically demanding housework (e.g. cleaning, tidying up)	On ….. days during the 4 weeks	Appr.. ….. minutes per day	Not done
Physically demanding care work (e.g. looking after children, caring for the sick)	On ….. days during the 4 weeks	Appr.. ….. minutes per day	Not done

<table>
<tr><td rowspan="7">Physical Activity and Sports Questionnaire (BSA)</td><td>4</td><td colspan="3">How many days and for how long did you engage in the following activities in the last 4 weeks?</td></tr>
<tr><td>Climbing stairs</td><td>On ….. days during the 4 weeks</td><td>Appr.. ….. floors per day</td><td>Not done</td></tr>
<tr><td>5</td><td colspan="3">Have you been doing regular exercise in the last 4 weeks?</td></tr>
<tr><td colspan="2">Yes – continue with Question 6</td><td colspan="2">No – Questionnaire completed</td></tr>
<tr><td>6</td><td colspan="3">What sporting activity or activities have you been doing?</td></tr>
<tr><td colspan="2">A
…………………………
(please enter here)</td><td>B
…………………………
(please enter here)</td><td>C
…………………………
(please enter here)</td></tr>
<tr><td colspan="2">I have done activity A
appr. ..... times in the last 4 weeks,
each time for
appr. ..... minutes</td><td>I have done activity B
appr. ..... times in the last 4 weeks,
each time for
appr. ..... minutes</td><td>I have done activity C
appr. ..... times in the last 4 weeks,
each time for
appr. ..... minutes</td></tr>
<tr><td>Pick up on interests in exercise and openness</td><td colspan="4">• Which types of exercise and sports did you enjoy most in the past? Why? What about them?
• Which types of exercise or sports interest you most at the moment? Which types of exercise or sports could you most likely imagine doing more often in the near future?
• Is there anything you've always wanted to try but haven't had the opportunity to do so yet?
• How open are you to trying new forms of exercise?
• What motivates you to try new things? Are there any factors that might prevent you from doing so?</td></tr>
<tr><td>Communicate homework</td><td colspan="4">Complete the Berner Motive and Goal Inventory questionnaire in preparation for the next conversation.: https://bmzi.ispw.unibe.ch/</td></tr>
<tr><td>Arrange a date for the next coaching</td><td colspan="4">Find a date for the next coaching (next week) based on the availability of the coach and coachee.
Briefly summarise what was discussed and provide information about what we can discuss in the next coaching (results of the questionnaire, planning the first physical activity session).</td></tr>
<tr><td>Follow-up</td><td colspan="4">Documentation of coaching in excel table:
• Duration: Preparation, Conversation, Follow-up
• Communication Chanels
• Interests in Physical Activity
• If applicable, transfer the following values from Oura: Average number of steps over the last 7 days, average number of minutes of light, moderate and intense physical activity</td></tr>
</table>

	Send homework and the next appointment to the coachee, e.g.:<br>*Subject: Homework and appointment confirmation*<br>*Dear [first name],*<br>*Thank you very much for the pleasant introductory coaching session.*<br>*As discussed, I would like to ask you to complete the following questionnaire over the next few days (before our next coaching session) and bring the answers with you to our next session. These will serve as the basis for the next session.*<br>*Questionnaire on sports-related motives and goals*<br>*Our next appointment will take place on [date, time] at [your home].*<br>*I am very much looking forward to continuing to work with you.*<br>*Best regards,*<br>*[First name]*

<table>
<tr><td colspan="2">Coaching 2 : BMZI and planning</td></tr>
<tr><td>Aims:</td><td>• Build and strengthen the relationship
• Discuss BMZI evaluation
• Plan visit to first exercise program
• Possibly already planning further visits to exercise programs
• Create framework conditions for physical activity</td></tr>
<tr><td>Duration</td><td>60min</td></tr>
<tr><td>Title</td><td>Content</td></tr>
<tr><td>Preparation</td><td>Extract the following values from Oura and transfer them to excel table:
• Average number of steps taken in the last 7 days
• Average number of minutes of light physical activity
• Average number of minutes of moderate physical activity
• Average number of minutes of intense physical activity
View notes from the last coaching.</td></tr>
<tr><td colspan="2">Conversation</td></tr>
<tr><td>Getting started/Arriving</td><td>Ask about past week:
• How did you feel after the first Coaching?
• How was your week?
Briefly refer to the last conversation and lead to the homework assignment
• How was the homework for you?</td></tr>
<tr><td>Discussion on BMZI evaluation</td><td>What was the outcome of the questionnaire?
What do you think of the result?
Last time you listed X, Y and Z as your interests in exercise. How do you classify the results of the questionnaire?
The aim of our collaboration is for you to enjoy exercise and find an exercise program that you would like to attend in the long term. What can you imagine? How much should the offer cost?</td></tr>
<tr><td>Planning first exercise class visit</td><td>Plan together for next week/next coaching session to attend the first exercise program. Register with the trainer. Discuss what is needed to participate in the offer:
- How do you get there?
- What do you wear?
- What do you need to take with you? (Water bottle, towel, sports shoes etc.)
- Who will look after your children?
- What could get in the way? And how do we make sure that I, as the coach, am not standing there alone next week?
Agree a meeting point and time, possibly at the coachee's home to walk the route together or directly before the exercise program.</td></tr>
</table>

If applicable, plan further visits to exercise classes	Depending on how much the coachee already knows about the direction their exercise program should take, you can plan the next visit straight away.<br>Otherwise, you will use the time in coaching 4 to reflect on activity 1 and plan another activity visit.
Creating a framework for physical activity	*What else needs to happen so you are more likely to exercise?*<br>*Do we need reminders to take the bike in the morning instead of the car? Or to walk one stop before getting on the streetcar, etc.?*<br>*Or do you need a rain jacket/trousers?*<br>*Do you need the right social support?*
Communicate homework	Preparation of the first attendance in exercise class, e.g.:<br>- Organize childcare<br>- Registration with trainer<br>- Entry in diary incl. reminder<br>- Prepare sportswear<br>- Pack bag<br>- Etc.
Arrange a date for the next coaching	The date of the next coaching session corresponds to the date of the exercise program or the meeting point or time that you have arranged to attend the exercise program.<br>Briefly summarize what was discussed and provide information about the next appointment.
**Follow-up**	Documentation of coaching in excel table:<br>• Duration: Preparation, Conversation, Follow-up<br>• Evaluation of BMZI<br>• Planning of first attendance of exercise session<br>Send homework and the next appointment to the coachee, e.g.:<br>*Subject: Homework and appointment confirmation*<br>*Dear [first name],*<br>*Thank you very much for the motivated second Coaching.*<br>*As discussed, I would ask you to prepare [X, Y and Z] so that we can attend [the yoga class] under the best possible conditions. I am very much looking forward to our first movement session together.*<br>*Our next appointment will be on [date, time] at [yoga studio XY].*<br>*I am looking forward to it.*<br>*Best regards*<br>*[First name]*

<table>
<tr><td colspan="2">Coaching 3 : Attendance of exercise session</td></tr>
<tr><td>Aims:</td><td>Joint attendance of a exercise session
Survey of the FS, FAS and Borg Scale
Reflection on the exercise experience
Evaluation of whether this activity will be continued in the future</td></tr>
<tr><td>Duration</td><td>60min</td></tr>
<tr><td>Title</td><td>Content</td></tr>
<tr><td>Preparation</td><td>Extract the following values from Oura and transfer them to excel table:
Average number of steps taken in the last 7 days
Average number of minutes of light physical activity
Average number of minutes of moderate physical activity
Average number of minutes of intense physical activity
View notes from the last coaching.

FS, FAS and Borg Scale ready for the mid-term and end of coaching query.</td></tr>
<tr><td colspan="2">Conversation</td></tr>
<tr><td>Getting started/Arriving</td><td>Ask about past week:
How did you feel after the last Coaching?
How was your week?
Briefly refer to the last conversation and lead to the feelings towards the forthcoming exercise session.
How did you prepare for your visit to the exercise program?
How do you feel now?
Would you be here if we hadn't made an appointment?</td></tr>
<tr><td>Participation in exercise session</td><td>At half-time and at the end, collect FS, FAS and Borg Scale:</td></tr>
<tr><td>Communicate homework</td><td>Answer/record self-reflection in the diary</td></tr>
<tr><td>Termin für nächstes Conversation vereinbaren</td><td>Find a date for the next coaching (next week). Either to attend another exercise program or for reflection.

Briefly summarize what was discussed and provide information about the next appointment.</td></tr>
<tr><td>Follow-up</td><td>Documentation of coaching in excel table:
Duration: Preparation, Conversation, Follow-up
FS, FAS, Borg of the first attended exercise session
Reflection of Exercise experience from coaches’ point of view
Send homework and the next appointment to the coachee, e.g.:

Subject: Homework and appointment confirmation

Dear [first name],</td></tr>
</table>

	Congratulations on your participation in the exercise program [XY].<br><br>*It is worth reflecting on the positive and negative aspects of the exercise program in a timely manner. You are welcome to use the diary with the reflection questions for this purpose. This will help you to reflect during our Reflection Coaching in [1 or two weeks]. Be honest. You will not be graded, but we will try to improve the next experience based on your experience.*<br><br>*[For the next session, I ask you to prepare [X, Y and Z] so that we can attend [the yoga class] in the best possible conditions. I am very much looking forward to our next movement session together].*<br><br>*Our next session will be on [date, time] at [yoga studio XY].*<br><br>*I am looking forward to it.*<br><br>*Best regards*<br><br>*[First name]*

<table>
<tr><td colspan="2">Coaching 4 : Reflection on exercise programmes</td></tr>
<tr><td>Aims:</td><td>Reflection: visit(s) to exercise programme(s)
Planning: future physical activity
Creating a framework for physical activity</td></tr>
<tr><td>Duration</td><td>60min</td></tr>
<tr><td>Title</td><td>Content</td></tr>
<tr><td>Preparation</td><td>Extract the following values from Oura and transfer them to excel table:
Average number of steps taken in the last 7 days
Average number of minutes of light physical activity
Average number of minutes of moderate physical activity
Average number of minutes of intense physical activity
View notes from the last coaching.</td></tr>
<tr><td colspan="2">Conversation</td></tr>
<tr><td>Getting started/Arriving</td><td>Ask about past week:
How did you feel after the last Coaching?
How was your week?
Briefly refer to the last conversation and lead to the reflection of the exercise session.
What was your first thought after the exercise programme with regard to continuing it?
How did your feelings about the exercise programme develop afterwards?</td></tr>
<tr><td>Reflection on exercise experience</td><td>How much did you like the exercise programme (scale 1-10)?

How did you like the form of exercise itself?

How did you like the setting (group, trainer, location, music)?

What was the best moment? Which emotion was particularly present? How did your body feel?

What did you like? Why did you like it?

What did you not like? Why?

What did you like, even if it was [strenuous or other negative quality]?

How did the exercise affect the rest of your day?

What does this mean for your future physical activity? Can you imagine attending the programme again? How would it need to be adapted so that you could enjoy it in the long term? What criteria should an alternative fulfil?</td></tr>
<tr><td>Create framework conditions for physical activity</td><td>What needs to happen so that even fewer obstacles stand in the way of exercising or so that exercising becomes more attractive/desirable?</td></tr>
</table>

<table>
<tr><td>Planning visit to next exercise session</td><td>Plan the next exercise programme together for next week/next coaching session. If you already enjoyed the previous exercise programme, it can of course be the same one again. Register with the trainer. Discuss what you need to participate in the programme:
- How do you get there?
- What do you wear?
- What do you need to take with you? (Water bottle, towel, sports shoes etc.)
- Who will look after your children?
- What could get in the way? And how do we make sure that I, as the coach, am not standing there alone next week?
Agree a meeting point and time, possibly at the coachee's home to walk the route together or directly before the exercise programme.</td></tr>
<tr><td>Create framework conditions for physical activity</td><td>What else needs to happen so that you are more likely to exercise?
Do you need reminders to take the bike in the morning instead of the car? Or to walk one stop before getting on the tram, etc.?
Or do you need a rain jacket/trousers?
Do you need the right social support?</td></tr>
<tr><td>Communicate homework</td><td>Preparation of the first attendance in exercise class, e.g.:
- Organize childcare
- Registration with trainer
- Entry in diary incl. reminder
- Prepare sportswear
- Pack bag
- Etc.</td></tr>
<tr><td>Arrange a date for the next coaching</td><td>The date of the next coaching session corresponds to the date of the exercise program or the meeting point or time that you have arranged to attend the exercise program.
Briefly summarize what was discussed and provide information about the next appointment.</td></tr>
<tr><td>Follow-up</td><td>Documentation of Coaching in excel table:
• Duration: Preparation, Conversation, Follow-up
• Reflection: Exercise session visit
• Plan for next exercise session visit
Send homework and the next appointment to the coachee, e.g.:
Subject: Homework and appointment confirmation
Dear [first name],
Thank you for your honest reflection in the Coaching today.
As discussed, I ask you to prepare [X, Y and Z] so that we can attend [the yoga class] under the best possible conditions. I am very much looking forward to our movement session together.
Our next appointment will be on [date, time] at [yoga studio XY].</td></tr>
</table>

	I am looking forward to it.<br>*Best regards*<br>*[First name]*

Coaching 8 : Note on the study; reflection or visit of exercise session		
	Reflection	Visit/Attendance
**Aims:**	• Reflection: visit(s) to exercise programme(s)<br>• Planning: future physical activity<br>• Creating a framework for physical activity	• Joint attendance of a exercise session<br>• Survey of the FS, FAS and Borg Scale<br>• Reflection on the exercise experience<br>• Evaluation of whether this activity will be continued in the future
**Duration**	60min	60min
**Title**	**Content**	**Content**
**Preparation**	Extract the following values from Oura and transfer them to excel table:<br>• Average number of steps taken in the last 7 days<br>• Average number of minutes of light physical activity<br>• Average number of minutes of moderate physical activity<br>• Average number of minutes of intense physical activity<br>View notes from the last coaching.	Extract the following values from Oura and transfer them to excel table:<br>• Average number of steps taken in the last 7 days<br>• Average number of minutes of light physical activity<br>• Average number of minutes of moderate physical activity<br>• Average number of minutes of intense physical activity<br>View notes from the last coaching.<br>FS, FAS and Borg Scale ready for the mid-term and end of coaching query.
**Conversation**		
Getting started/Arriving	Ask about past week:<br>• *How did you feel after the last Coaching?*<br>• *How was your week?*<br>Briefly refer to the last conversation and lead to the reflection of the exercise session.<br>• *What was your first thought after the exercise programme with regard to continuing it?*<br>*How did your feelings about the exercise programme develop afterwards?*	Ask about past week:<br>• *How did you feel after the last Coaching?*<br>• *How was your week?*<br>Briefly refer to the last conversation and lead to the feelings towards the forthcoming exercise session.<br>• *How did you prepare for your visit to the exercise program?*<br>• *How do you feel now?*<br>*Would you be here if we hadn't made an appointment?*
Main part	*How much did you like the exercise programme (scale 1-10)?*	Collect FS, FAS and Borg Scale at half-time and at the end.

	How did you like the form of exercise itself?<br><br>*How did you like the setting (group, trainer, location, music)?*<br><br>*What was the best moment? Which emotion was particularly present? How did your body feel?*<br><br>*What did you like? Why did you like it?*<br><br>*What did you not like? Why?*<br><br>*What did you like, even if it was [strenuous or other negative quality]?*<br><br>*How did the exercise affect the rest of your day?*<br><br>*What does this mean for your future physical activity? Can you imagine attending the programme again? How would it need to be adapted so that you could enjoy it in the long term? What criteria should an alternative fulfil?*	
	What needs to happen so that even fewer obstacles stand in the way of exercising or so that exercising becomes more attractive/desirable?	
	Plan the next exercise programme together for next week/next coaching session. If you already enjoyed the previous exercise programme, it can of course be the same one again. Register with the trainer. Discuss what you need to participate in the programme:<br><br>- *How do you get there?*<br>- *What do you wear?*<br>- *What do you need to take with you? (Water bottle, towel, sports shoes etc.)*<br>- *Who will look after your children?*<br>- *What could get in the way? And how do we make sure that I, as the coach, am not standing there alone next week?*<br>- Agree a meeting point and time, possibly at the coachee's home to walk the	

	route together or directly before the exercise programme.	
	What else needs to happen so that you are more likely to exercise?<br>*Do you need reminders to take the bike in the morning instead of the car? Or to walk one stop before getting on the tram, etc.?*<br>*Or do you need a rain jacket/trousers?*<br>*Do you need the right social support?*	
Communicate homework	Preparation of the first attendance in exercise class, e.g.:<br>- Organize childcare<br>- Registration with trainer<br>- Entry in diary incl. reminder<br>- Prepare sportswear<br>- Pack bag<br>- Etc.	Answer/record self-reflection in the diary
Note on the study	*You have been in the study now for 8 weeks and the next measurement is already due.*<br>*Have you already been contacted by the study management? If not, you can assume that contact will be made soon to arrange the appointment.*	
Arrange a date for the next coaching	The date of the next coaching session corresponds to the date of the exercise program or the meeting point or time that you have arranged to attend the exercise program.<br>Briefly summarize what was discussed and provide information about the next appointment.	Find a date for the next coaching (next week). Either to attend another exercise program or for reflection.<br>Briefly summarize what was discussed and provide information about the next appointment.
**Follow-up**	Documentation of Coaching in excel:<br>• Duration: Preparation, Conversation, Follow-up<br>• Reflection: Exercise session visit<br>• Plan for next exercise session visit<br>Send homework and the next appointment to the coachee, e.g.:<br>*Subject: Homework and appointment confirmation*<br>*Dear [first name],*	Documentation of coaching in excel table:<br>• Duration: Preparation, Conversation, Follow-up<br>• FS, FAS, Borg of the first attended exercise session<br>• Reflection of Exercise experience from coaches' point of view<br>Send homework and the next appointment to the coachee, e.g.:

	Thank you for your honest reflection in the Coaching today.<br><br>*As discussed, I ask you to prepare [X, Y and Z] so that we can attend [the yoga class] under the best possible conditions. I am very much looking forward to our movement session together.*<br><br>*Our next appointment will be on [date, time] at [yoga studio XY].*<br><br>*I am looking forward to it.*<br><br>*Best regards*<br><br>*[First name]*	*Subject: Homework and appointment confirmation*<br><br>*Dear [first name],*<br><br>*Congratulations on your participation in the exercise program [XY].*<br><br>*It is worth reflecting on the positive and negative aspects of the exercise program in a timely manner. You are welcome to use the diary with the reflection questions for this purpose. This will help you to reflect during our Reflection Coaching in [1 or two weeks]. Be honest. You will not be graded, but we will try to improve the next experience based on your experience.*<br><br>*[For the next session, I ask you to prepare [X, Y and Z] so that we can attend [the yoga class] in the best possible conditions. I am very much looking forward to our next movement session together].*<br><br>*Our next session will be on [date, time] at [yoga studio XY].*<br><br>*I am looking forward to it.*<br><br>*Best regards*<br><br>*[First name]*

<table>
<tr><th colspan="2">Coaching 10 : Conclusion</th></tr>
<tr><td>Aims:</td><td>Rounding off the coaching process or, in the case of IG2, planning the continuation
Clarify final questions
Sending into the future with self-confidence</td></tr>
<tr><td>Duration</td><td>60min</td></tr>
<tr><td>Title</td><td>Content</td></tr>
<tr><td>Preparation</td><td>Extract the following values from Oura and transfer them to excel table:
Average number of steps taken in the last 7 days
Average number of minutes of light physical activity
Average number of minutes of moderate physical activity
Average number of minutes of intense physical activity
View notes from the last coaching. What else is needed so that the coachee can continue independently?</td></tr>
<tr><td colspan="2">Conversation</td></tr>
<tr><td>Getting started/Arriving</td><td>Ask about past week:
How did you feel after the last Coaching?
How was your week?
Briefly refer to the last conversation and lead to the last coaching session (IG1) or to the change of setting (IG2):
IG1: Today is our last coaching session together. That's why it's important to me that I can answer your questions and give you what you still need to continue on your own.
IG2: Today we have the last coaching session of the first part together. After that, we will switch to shorter follow-up conversations, which we will conduct by telephone. It is therefore important to me that we discuss today what you still need in order to move on to the next coaching phase.</td></tr>
<tr><td>Review</td><td>Let's look back on the last 10 weeks together. A lot has happened.
What was your biggest success or key moment?
What will you take with you for the future?
What are you proud of?
What is your feedback on the process?</td></tr>
<tr><td>Outlook into the future</td><td>What else do you need so that you can continue successfully?
What support do you still need from me so that you can get off to a good start with “self-coaching”?
What will you focus on in the coming days, weeks and months?
What is your plan for the next few days and weeks?
What is your next milestone?
What barriers might arise in the future? What strategies do you have to overcome them?</td></tr>
</table>

**IG2**: Outlook Remote Coaching	*From now on, we will meet briefly on the phone every other week (20 minutes) to ensure that you keep moving in the long term. The idea is that you continue to attend exercise class Y X times a week and we use the conversation to clarify your questions, overcome any hurdles that may arise and/or make further adjustments to your exercise behavior.*<br>*How does that sound to you?*<br>*What is your plan until our next Conversation? What are you planning to do?*
**IG2:** Communication channel	Arrangement of communication channels<br>• Channel for making appointments?<br>• Channel for communicating agreements?<br>• Channel for telephone conversations?<br>*“If you are unable to attend an agreed appointment, please let me know as early as possible (by text message or other arrangement) so that we can reschedule the appointment.”*
Self-Monitoring/ Oura Ring	*You will now continue to wear the ring for another 6 months. What do you like best about the ring? How will you use it to support your behavior change?*
**IG2:** Communicate homework	Depends on agreement above.
**IG2**: Arrange a date for the next coaching	Find a date for the next conversation (in 2 weeks) according to the availability of coach and coachee.<br>Briefly summarize what was discussed and inform about the next appointment.
**IG1:** Goodbye	*What does it look like? Can we finalize our collaboration at this point? Do you feel ready to be your own coach?*<br>*It has been a great pleasure to accompany you on your journey. Thank you for your trust and the wonderful time. I wish you all the best.*
**Follow-up**	Documentation of Cocahing in excel table:<br>• Duration: Preparation, Conversation, Follow-up<br>• Review of coaching process<br>• Outlook into self-coaching resp. cognition-based coaching<br>**IG1: Sending coaching completion mail**<br>*Subject: Coaching Completion*<br>*Dear [first name],*<br>*Thank you very much for the appreciative coaching session today.*<br>*I am very pleased that you have achieved [success]. I hope that you can continue to benefit from our work together for a long time to come and that you enjoy exercising.*<br>*Thank you for your trust and the wonderful collaboration.*<br>*All the best*<br>*[First name]*

	**IG2: Sending the homework assignment and the next appointment to the coachee**<br><br>*Subject: Homework and appointment confirmation*<br><br>*Dear [first name],*<br><br>*Thank you for the open coaching conversation today.*<br><br>*We are now switching to the bi-weekly telephone follow-up coaching. You have set yourself [X, Y and Z] until the next appointment. I'm really looking forward to hearing about your experiences.*<br><br>*Our next appointment will take place on [date, time] by telephone. I will call you from [own telephone number].*<br><br>*I am looking forward to it.*<br><br>*Best regards*<br><br>*[First name]*

3 Cognition-based follow-up coachings

**Coaching 11: Setting goals**	
**Aims:**	• Clarification of goals and mandate for further cooperation<br>• Determination of actual state<br>• Barrier management and action planning
**Duration**	20-30min
**Title**	**Content**
**Preparation**	Extract the following values from Oura and transfer them to excel table:<br>• Average number of steps taken in the last 7 days<br>• Average number of minutes of light physical activity<br>• Average number of minutes of moderate physical activity<br>• Average number of minutes of intense physical activity<br>View notes from the last coaching.
**Conversation**	
Getting started/Arriving	Ask about past two weeks:<br>• *How did you feel after the last Coaching?*<br>• *How was the time*<br>Briefly refer to the last conversation and lead to the new form of coaching via telephone.
Clarify information on the process/framework conditions	*Perhaps you remember our first meeting. In the second part of this coaching programme, we will now work on keeping you moving in the long term. Together we will define goals, overcome obstacles and establish habits. We have already laid the foundations for this in the last 10 sessions. Now it's time for sustainability.*<br>*We talk on the phone every 2 weeks on average. We look at how you continue to succeed in getting moving and what else is needed so that you can keep moving in the long term. The conversations will last between 20 and 30 minutes. If there is less to discuss, we can also hang up after 5 minutes. We do it the way that suits you best.*<br>*It is important to me that we make the appointments as binding as before so that I can plan my responsibilities.*
Communication channel	*In Part 1, we used X as the communication channel. Is that still the case for you?*
Clarification of task/goals	*So far you go to [exercise programme] X times a week. Apart from that, you exercise at [work, transport, with children, etc.]. How satisfied are you with your level of activity?*<br>*Where would you like to be in six months' time in terms of your exercise and fitness? Why? How realistic do you think it is to achieve this in 6 months? How motivated are you to achieve this?*<br>*What support do you expect from me to help you achieve your goal? What is my task? What needs to change for you to be there in 6 months' time?*

Partial Tasks	Define, what the first step towards achieving the goal, is.
Determination of actual state by means of scaling questions and/or Oura values etc.	Perhaps you would like to record a few values together in order to compare them in the middle and at the end of the cognition-based coaching?<br><br>Scaling questions, e.g:<br><br>• How active do you feel in everyday life?<br>• (1 = hardly active, 10 = very active)<br><br>• How regularly do you exercise each week (e.g. walking, cycling, etc.)?<br>• (1 = very irregular, 10 = daily and constant)<br><br>• How much do you enjoy exercise?<br>• (1 = none at all, 10 = great pleasure)<br><br>• How much time per week do you invest in physical activity?<br>• (1 = <30 minutes, 10 = >7 hours)<br><br>• How well do you manage to integrate exercise into your everyday life?<br>• (1 = very difficult, 10 = completely natural)<br><br>• How would you rate your general physical fitness?<br>• (1 = very low, 10 = excellent)<br><br>• What is your current level of endurance? (e.g. when walking, jogging, cycling)<br>• (1 = very poor, 10 = top fit)<br><br>• How is your strength or muscle power in everyday life or training?<br>• (1 = barely there, 10 = very well developed)<br><br>• How mobile or flexible do you feel?<br>• (1 = very immobile, 10 = very flexible)<br><br>• How quickly do you recover after physical exertion?<br>• (1 = takes a very long time, 10 = very quickly and easily)<br><br>Or number of steps. If necessary, make your goals measurable with the help of the scaling questions and other values.
Action planning	Based on this, determine what the action plan(s) should look like.

Barrier management	What barriers have already arisen in the past? What barrieres could still come up?<br>How can they be overcome?
Communicate homework	
Arrange a date for the next coaching	Find a date for the next conversation (in 2 weeks) according to the availability of coach and coachee.<br>Briefly summarize what was discussed and inform about the next appointment.
**Follow-up**	Documentation of the coaching in excel table:<br>• Duration: Preparation, Conversation, Follow-up<br>• BCTs documentation<br><br>Sending the homework and the next appointment to the coachee, e.g:<br>*Subject: Homework and appointment confirmation*<br>*Dear [first name],*<br>*Thank you for the successful telephone coaching conversation.*<br>*As discussed, you will gain experience with [X, Y and Z] over the next two weeks. In the next conversation we will reflect on your experiences and make adjustments to the plan where necessary. I am very much looking forward to hearing from you again.*<br>*Our next appointment will take place on [date, time] by phone. I will call you at the agreed time.*<br>*I'm looking forward to it.*<br>*Best regards*<br>*[first name]*

**Coaching 12-16 und 18-21: Individual use of BCTs**	
**Aims:**	• According to individual status and needs<br>• Ensure sustainability of the increase in movement
**Duration**	20-30min
**Title**	**Content**
**Preparation**	Extract the following values from Oura and transfer them to excel table:<br>• Average number of steps taken in the last 7 days<br>• Average number of minutes of light physical activity<br>• Average number of minutes of moderate physical activity<br>• Average number of minutes of intense physical activity<br>View notes from the last coaching.
**Conversation**	
Getting started/Arriving	Ask about past two weeks:<br>• *How did you feel after the last Coaching?*<br>• *How was the time*<br>Briefly refer to the last conversation and lead to the homework.<br>• *How was the homework for you?*
Define a topic for today according to individual needs, e.g:	Difficulties in implementation or anticipation of difficulties? What are the reasons for it not being implemented?<br>• Everyday life is too stressful: Action planning/barrier management: *When could exercise still find a place?*<br>• Motivation decreases: Rewarding/focussing on past successes: *What have you already achieved/how could you reward yourself?*<br>• Movement is forgotten: Stimuli and cues/habit formation: *Is there anything that could remind you of exercise/how could exercise become a regular habit?*<br>• Celebrating success: Feedback on behaviour/reward: *What was your highlight/how did you do it?*<br>• Lack of social support: Social support: *Is there someone who would accompany you?*<br>• Limited time resources: Action planning/barrier management: *If you don't have much time - which mini-exercise unit is still suitable/what is realistically possible in stressful weeks?*<br>• Relapse/longer break: focussing on past successes/change of perspective: *how did you manage it last time/what did you still do well during this phase?*<br>• Goal seems too far away/frustration: Behavioural Aims/Subtasks: *Do we want to divide the big goal into smaller Aims? What would be a first mini-success by next week?*<br>• Movement = duty instead of pleasure: Change of perspective: *What about movement has felt good so far/how could we organise it so that it feels easier, freer?*<br>• Motivation boost, but no structure: Goal setting/action planning: *Shall we make a plan together while you are motivated/how can you utilise the energy of this week?*

	• Etc.
Adjustment of targets and plans if necessary	
Communicate homework	
Arrange a date for the next coaching	Find a date for the next conversation (in 2 weeks) according to the availability of coach and coachee.<br>Briefly summarize what was discussed and inform about the next appointment.
**Follow-up**	Documentation of the coaching in excel table:<br>• Duration: Preparation, Conversation, Follow-up<br>• BCTs documentation<br><br>Sending the homework and the next appointment to the coachee, e.g:<br>*Subject: Homework and appointment confirmation*<br>*Dear [first name],*<br>*Thank you for the successful telephone coaching conversation.*<br>*As discussed, you will gain experience with [X, Y and Z] over the next two weeks. In the next conversation we will reflect on your experiences and make adjustments to the plan where necessary. I am very much looking forward to hearing from you again.*<br>*Our next appointment will take place on [date, time] by phone. I will call you at the agreed time.*<br>*I'm looking forward to it.*<br>*Best regards*<br>*[first name]*

**Coaching 17: Interim status**	
**Aims:**	• According to individual status and needs<br>• Ensure sustainability of the increase in movement<br>• Raise interim status
**Duration**	20-30min
**Title**	**Content**
**Preparation**	Extract the following values from Oura and transfer them to excel table:<br>• Average number of steps taken in the last 7 days<br>• Average number of minutes of light physical activity<br>• Average number of minutes of moderate physical activity<br>• Average number of minutes of intense physical activity<br>View notes from the last coaching.
**Conversation**	
Getting started/Arriving	Ask about past two weeks:<br>• *How did you feel after the last Coaching?*<br>• *How was the time*<br>Briefly refer to the last conversation and lead to the homework.<br>• *How was the homework for you?*
Raise interim status/repeat determination of actual state	Copy scaling questions and comparison values (steps etc.) here and compare them.
Adjustment of targets and plans if necessary	
Communicate homework	
Arrange a date for the next coaching	Find a date for the next conversation (in 2 weeks) according to the availability of coach and coachee.<br>Briefly summarize what was discussed and inform about the next appointment.
**Follow-up**	Documentation of the coaching in excel table:<br>• Duration: Preparation, Conversation, Follow-up<br>• BCTs documentation<br><br>Sending the homework and the next appointment to the coachee, e.g:<br>*Subject: Homework and appointment confirmation*<br>*Dear [first name],*<br>*Thank you for the successful telephone coaching conversation.*

	As discussed, you will gain experience with [X, Y and Z] over the next two weeks. In the next conversation we will reflect on your experiences and make adjustments to the plan where necessary. I am very much looking forward to hearing from you again.<br><br>*Our next appointment will take place on [date, time] by phone. I will call you at the agreed time.*<br><br>*I'm looking forward to it.*<br><br>*Best regards*<br><br>*[first name]*

<table>
<tr><th colspan="2">Coaching 22: Preparation of end of coaching</th></tr>
<tr><td>Aims:</td><td>According to individual status and needs
Ensure sustainability of the increase in movement</td></tr>
<tr><td>Duration</td><td>20-30min</td></tr>
<tr><td>Title</td><td>Content</td></tr>
<tr><td>Preparation</td><td>Extract the following values from Oura and transfer them to excel table:
Average number of steps taken in the last 7 days
Average number of minutes of light physical activity
Average number of minutes of moderate physical activity
Average number of minutes of intense physical activity
View notes from the last coaching.</td></tr>
<tr><td colspan="2">Conversation</td></tr>
<tr><td>Getting started/Arriving</td><td>Ask about past two weeks:
How did you feel after the last Coaching?
How was the time
Briefly refer to the last conversation and lead to the homework.
How was the homework for you?</td></tr>
<tr><td>Preparation of end of coaching</td><td>Today is our second last Coaching. It is important to me that you are optimally prepared to maintain your exercise programme as discussed also after the coaching.
What else do you need so that you can continue successfully after our collaboration?
What other support do you need instead of me so that you can get off to a good start with ‘self-coaching’?
What barriers might arise in the future? What strategies do you have to overcome them?
What will you focus on in the coming days, weeks and months?
What is your plan for the next few days and weeks?
What is your next milestone?</td></tr>
<tr><td>Note on the study</td><td>You have now been in the study for 7.5 months and the next and final measurement is already due.
Have you already been contacted by the study management? If not, you can assume that contact will be made soon to arrange the appointment.</td></tr>
<tr><td>Communicate homework</td><td></td></tr>
<tr><td>Arrange a date for the next coaching</td><td>Find a date for the next conversation (in 2 weeks) according to the availability of coach and coachee.
Briefly summarize what was discussed and inform about the next appointment.</td></tr>
</table>

**Follow-up**	Documentation of the coaching in excel table:<br>• Duration: Preparation, Conversation, Follow-up<br>• BCTs documentation<br><br>Sending the homework and the next appointment to the coachee, e.g:<br>*Subject: Homework and appointment confirmation*<br>*Dear [first name],*<br>*Thank you for the successful telephone coaching conversation.*<br>*As discussed, you will gain experience with [X, Y and Z] over the next two weeks. In the next conversation we will reflect on your experiences and make adjustments to the plan where necessary. I am very much looking forward to hearing from you again.*<br>*Our next appointment will take place on [date, time] by phone. I will call you at the agreed time.*<br>*I'm looking forward to it.*<br>*Best regards*<br>*[first name]*

Coaching 23 : Last coaching - conclusion	
**Aims:**	• Rounding off the coaching process<br>• Clarify final questions<br>• Sending you into the future with confidence
**Duration**	20-30min
**Title**	**Content**
**Preparation**	Extract the following values from Oura and transfer them to excel table:<br>• Average number of steps taken in the last 7 days<br>• Average number of minutes of light physical activity<br>• Average number of minutes of moderate physical activity<br>• Average number of minutes of intense physical activity<br>View notes from the last coaching. What else is needed so that the coachee can continue independently?
**Conversation**	
Getting started/Arriving	Ask about past two weeks:<br>• *How did you feel after the last Coaching?*<br>• *How was the time?*<br>Briefly refer to the last conversation and lead to the last coaching session.<br>• *Today is our last coaching session together. That's why it's important to me that I can answer your questions and give you what you still need to continue on your own.*
Review	*Let's look back together on the last 6-8 months. A lot has happened.*<br>*What was your biggest success or key moment?*<br>*What are you taking with you for the future?*<br>*What are you proud of?*<br>*What is your feedback on the process?*
Repeat assessment of actual state	Copy scaling questions and comparison values (steps etc.) here and compare them.
Outlook into the future	*What else do you need so that you can continue successfully?*<br>*What support do you need instead of me so that you can get off to a good start with 'self-coaching'?*<br>*What are you focussing on in the coming days, weeks and months?*<br>*What is your plan for the next few days and weeks?*<br>*What is your next milestone?*<br>*What barriers might arise in the future? What strategies do you have to overcome them?*
Goodbye	*What does it look like? Can we finalise our collaboration at this point? Do you feel ready to be your own coach?*

	It has been a great pleasure to accompany you on your journey. Thank you for your trust and the wonderful time. I wish you all the best.
**Follow-up**	Documentation of the coaching in excel table:<br>• Duration: Preparation, Conversation, Follow-up<br>• BCTs documentation<br>• Review of the coaching process<br>• Outlook on self-coaching or cognition-based coaching<br><br>Sending the homework and the next appointment to the coachee, e.g:<br><br>**Sending coaching completion mail**<br>*Subject: Coaching completion*<br>*Dear [first name],*<br>*Thank you very much for the appreciative coaching completion today.*<br>*I am very pleased that you have achieved [success]. I hope that you can continue to benefit from our collaboration for a long time to come and that you enjoy the exercise.*<br>*Thank you for your trust and the wonderful collaboration.*<br>*All the best*<br>*[first name]*
