# Supplementary material for: Lifestyle physical activity coaching in outpatients with major depressive disorder (PACOUTPAT): study protocol for a randomized controlled trial on physical activity, depression, and quality of life
Source: Trials. 2026 Feb 17;27:231. doi: 10.1186/s13063-026-09500-1 (PMC13014798; doi:10.1186/s13063-026-09500-1)
Supplement: Supplementary file 2 — Additional file 2: Timeline of PACOUTPAT-trial. [file 13063_2026_9500_MOESM2_ESM.pdf]

University of Basel

Department of
Sport, Exercise and Health

PACOUTPAT

# Timeline of PACOUTPAT-trial

| Milestones | 2025 | | | | 2026 | | | | 2027 | | | | 2028 | | | |
|---|---|---|---|---|---|---|---|---|---|---|---|---|---|---|---|---|
| | Q1 | Q2 | Q3 | Q4 | Q1 | Q2 | Q3 | Q4 | Q1 | Q2 | Q3 | Q4 | Q1 | Q2 | Q3 | Q4 |
| Ethics Approval | | | | | | | | | | | | | | | | |
| Recruitment | | | | | | | | | | | | | | | | |
| Baseline Assessments | | | | | | | | | | | | | | | | |
| Intervention | | | | | | | | | | | | | | | | |
| Post Assessments | | | | | | | | | | | | | | | | |
| Follow-up Assessments | | | | | | | | | | | | | | | | |
| Dissemination | | | | | | | | | | | | | | | | |
